# A Green Sol–Gel Route to Fe_3_O_4_@TiO_2_–CuO Photocatalysts with Structural Stability, Visible–Light Activity, and Magnetic Recoverability

**DOI:** 10.1021/acsomega.5c09939

**Published:** 2026-03-04

**Authors:** Gabriel Bardella Stelzer, Isabella C. Prescilio, Leonardo G. Vasconcelos, Andris F. Bakuzis, Marcos J. Jacinto

**Affiliations:** † Nano Materials and Catalysis Laboratory, Institute of Chemistry, Federal University of Mato Grosso, Cuiabá 78060-900, Brazil; ‡ Laboratory of Research in Natural Products Chemistry and New Synthetic Methodologies in Organic Chemistry, Institute of Chemistry, Federal University of Mato Grosso, Cuiabá 78060-900, Brazil; § Institute of Physics, Federal University of Goiás, Goiânia 74690-900, Brazil

## Abstract

In this work, we present the synthesis of a multifunctional Fe_3_O_4_@TiO_2_–CuO photocatalyst, integrating *Magonia pubescens* plant extract and urea-assisted pH modulation. The as-prepared composite exhibits outstanding visible-light photocatalytic performance, achieving 75% degradation of rhodamine B (RhB, 15 ppm) after 5 h under 100 W LED irradiation and retaining more than 45% activity across four reuse cycles enabled by simple magnetic recovery. Notably, urea-assisted synthesis reduces iron leaching by ∼50%, preserving magnetic integrity and enabling efficient catalyst separation. Comprehensive structural, morphological, and surface analyses (TEM, XRD, XPS, XRF, VSM, ICP, GC–MS) confirm the formation of a robust, magnetically recoverable hybrid with a narrowed band gap of 1.65 eV and enhanced stability. GC–MS analysis revealed progressive mineralization of RhB into low-molecular-weight intermediates.

## Graphical abstract

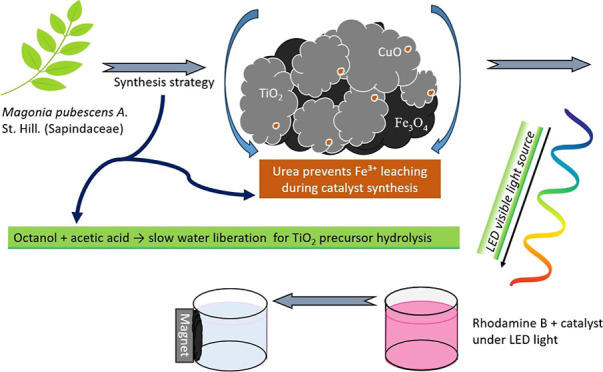

## 1 Introduction

The discharge of synthetic dyes into aquatic systems remains a major environmental and public-health concern, driven by their widespread use in textile, cosmetic, and pharmaceutical industries. RhB, a xanthene-class dye (C_28_H_31_ClN_2_O_3_; MW 479.02 g/mol), is particularly problematic due to its high solubility, chemical stability, and resistance to conventional treatment methods, factors linked to genotoxic, mutagenic, and carcinogenic effects even at trace levels. In addition, RhB elevates chemical and biological oxygen demand (COD/BOD) in aquatic environments, contributing to oxygen depletion and long-term ecological damage.^1–3^

Due to the limited effectiveness of biological and physicochemical treatments in removing persistent organic pollutants (POPs), advanced oxidation processes (AOPs) which generate highly reactive oxygen species (ROS), such as hydroxyl (^•^OH) and superoxide (^•^O_2_^–^) radicals for pollutant mineralization have been widely applied. Among these, photocatalysis, photoelectrocatalysis, and Fenton or Fenton-like reactions exhibit strong oxidation performance in wastewater treatment.^4,5^ Within this context, the development of visible-light-responsive and magnetically recoverable composites, such as Fe_3_O_4_@TiO_2_–CuO, emerges as a practical and environmentally benign alternative. In such systems, semiconductor-based catalysts are photoactivated under light irradiation, leading to the generation of reactive oxygen species (ROS), particularly hydroxyl radicals (^•^OH), which are capable of oxidizing and mineralizing complex organic pollutants into CO_2_ and H_2_O under ambient conditions. Titanium dioxide (TiO_2_), especially in its anatase phase, is one of the most widely used photocatalysts due to its low cost, high chemical stability, and strong oxidative power.^6,7^ However, the wide band gap of TiO_2_ (∼3.2 eV)^9^ restricts its photoactivity to the ultraviolet (UV) range, which accounts for less than 5% of the solar spectrum, significantly limiting its practical efficiency under natural sunlight.

To overcome this limitation, extensive efforts have focused on developing visible-light-responsive photocatalysts. A promising approach is coupling TiO_2_ with narrow-band gap semiconductors such as CuO (band gap ∼1.4 eV), which efficiently absorbs visible light.^8^ TiO_2_–CuO heterojunctions enhance charge separation via p–n junction formation, suppressing electron–hole recombination and improving visible-light photocatalytic activity. For example, Kubiak et al. reported that microwave-assisted TiO_2_–CuO heterojunctions exhibit significantly enhanced visible-light degradation efficiency and facile magnetic recovery,^9^ while Hamad et al. demonstrated that S-scheme CuO@TiO_2_ composites further improve organic pollutant degradation through efficient interfacial charge transfer.^10^ Despite these advantages, CuO alone exhibits rapid charge recombination and often requires oxidant additives such as H_2_O_2_ to sustain photocatalytic efficiency; therefore, synergistic integration with stable n-type oxides remains essential to maximize performance.

A further enhancement is achieved by incorporating magnetic components, such as magnetite (Fe_3_O_4_), into the photocatalyst structure. Fe_3_O_4_ exhibits superparamagnetic properties that allow for rapid and efficient catalyst recovery using external magnetic fields, an advantage for practical applications in wastewater treatment.^11^

Recent studies have demonstrated that Fe_3_O_4_@TiO_2_ core–shell structures can simultaneously deliver magnetic separability and high photocatalytic efficiency.^12,13^ Furthermore, Fe_3_O_4_ contributes to improved charge transport across the heterostructure, potentially enhancing interfacial electron transfer dynamics. These multifunctional systems, combining visible-light responsiveness, enhanced charge separation, and magnetic recoverability, represent a frontier in photocatalytic materials design.

Traditionally, metal oxide nanoparticles have been produced through sol–gel, hydrothermal/solvothermal, precipitation, microemulsion, and thermal decomposition methods. Although these techniques offer good control over size, crystallinity, and morphology, they often require high temperatures, toxic solvents or reducing agents, and multistep processing.^14^ Such requirements pose limitations for large-scale production, including environmental concerns, elevated energy use, and occupational safety issues (Scheme 1).

**Scheme 1:**
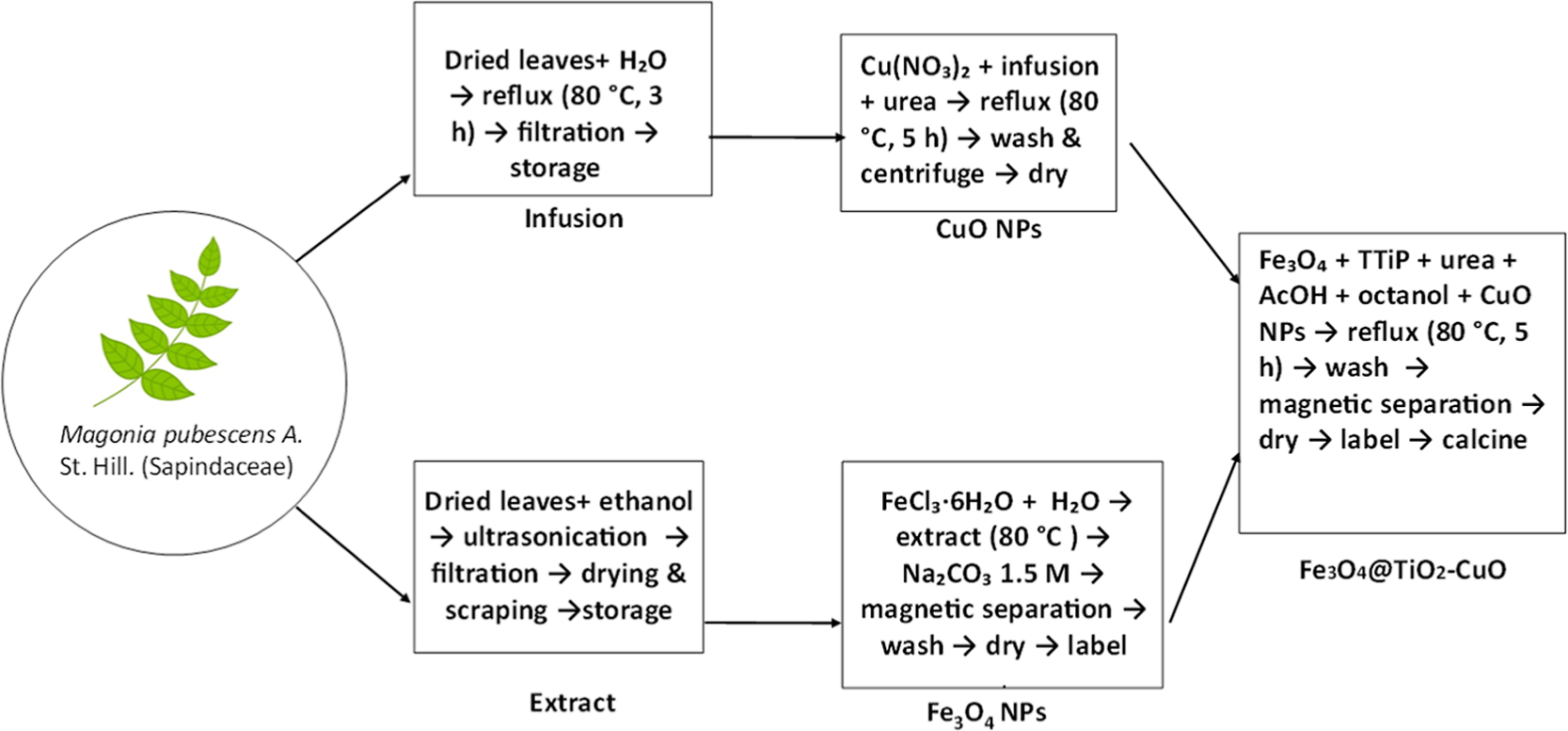
Flow Chart Summarizing the Synthesis of the Fe_3_O_4_@TiO_2_–CuO Composite, Including the Preparation of Precursors and Final Calcination Step

Recent trends in materials synthesis emphasize the adoption of green chemistry principles. Phytosyntheis, the use of plant extracts as both reducing and stabilizing agents, offers a sustainable and eco-friendly alternative.^15–18^

Plant-derived polyphenols, flavonoids, alkaloids, and organic acids can mediate the formation of metal or metal oxide nanoparticles under mild conditions, while simultaneously functionalizing the surface and enhancing colloidal stability. Numerous recent works have reported the successful biosynthesis of metal oxide-based photocatalysts using plant extracts, achieving comparable or superior performance to those synthesized by traditional routes. For example, Yitagesu et al. demonstrated the green synthesis of TiO_2_/CuO nanocomposites using *Impatiens tinctoria* leaf extract as a biogenic reducing and stabilizing agent, yielding polycrystalline nanoparticles with an average size of approximately 29 nm and a mesoporous surface area of 87.5 m^2^/g. Comprehensive structural and optical analyses, including PL and HRTEM, confirmed the formation of an effective heterojunction and a notable reduction in exciton recombination.^19^ The resulting nanocomposite exhibited outstanding photocatalytic performance, achieving 99% degradation of methylene blue under optimized conditions (40 min of reaction time), along with excellent reusability. Similarly, in a study conducted by our research group, Prescilio et al. reported the biosynthesis of magnetic FeOx@ZnO and FeOx@ZnO@NiO photocatalysts using *Magonia pubescens* extracts, achieving over 85% degradation of RhB under visible LED light after 180 min of reaction.^20^

The thermodecomposition of inexpensive organic compounds such as urea can simultaneously promote a controlled pH increase, enable sol–gel-like hydrolysis and condensation of metal precursors, and enhance interfacial adhesion between inorganic phases. When combined with plant-derived substrates rich in functional metabolites, this strategy also supports the bioassisted reduction and stabilization of nanoparticles, offering an environmentally benign route to hybrid nanostructures. Upon heating, urea releases ammonia and carbon dioxide, generating a gradual and homogeneous pH rise,^21–24^ which facilitates the controlled hydrolysis/condensation of oxide precursors and helps maintain the structural stability of magnetic components. Moreover, the evolving basic environment improves interfacial interactions between the forming oxide network and the Fe_3_O_4_ core. Urea also contributes to stabilizing the Fe^2+^/Fe^3+^ redox balance,^25^ preserving the magnetite phase and mitigating Fe^2+^ oxidation.

In this study, we report the synthesis of a multifunctional Fe_3_O_4_@TiO_2_–CuO photocatalyst via a urea-assisted sol–gel process under mild conditions. Urea acts as a pH modulator, gradually increasing alkalinity during thermal decomposition, while in situ water generated by esterification of glacial acetic acid and 1-octanol enables controlled hydrolysis of titanium isopropoxide. This combination promotes the formation of a stable TiO_2_ shell on green-synthesized Fe_3_O_4_ nanoparticles, with CuO immobilization further enhancing visible-light photocatalytic activity.

By integrating phytochemical-mediated synthesis with a controlled sol–gel approach involving urea-assisted pH modulation and esterification-driven hydrolysis, we established a sustainable and process-compatible route to the Fe_3_O_4_@TiO_2_–CuO hybrid, suitable for scalable and magnetically recoverable catalytic applications. Urea was shown to be critical in suppressing iron leaching from the Fe_3_O_4_ core, as its absence led to an approximately 2-fold increase in Fe concentration, underscoring the importance of controlled stabilization for effective TiO_2_ encapsulation and magnetic core preservation.

## 2 Experimental Procedure

The complete fabrication of the material is outlined in Scheme 1. Dried and powdered leaves of *M. pubescens* A. St. Hil. were collected locally. Ethanol (95%) and copper­(II) nitrate trihydrate were purchased from Dinâmica (Brazil). Iron­(III) chloride hexahydrate was obtained from Synth (Brazil), and sodium carbonate from Vetec (Brazil). Titanium­(IV) isopropoxide was supplied by Sigma-Aldrich (USA). Glacial acetic acid was purchased from Anidrol (Brazil), and 1-octanol from Êxodo Científica (Brazil). Distilled water was obtained from the laboratory purification system. All reagents were of analytical grade and used as received.

### 2.1 Synthesis of Fe_3_O_4_ Nanoparticles

Magnetic nanoparticles were synthesized following a previously reported method.^26^ An infusion was prepared by refluxing 5 g of dried *M. pubescens* leaves in 50 mL of distilled water at 80 °C for 3 h. The ethanolic extract was obtained by ultrasonication of ground leaves in ethanol for 30 min, followed by filtration, solvent evaporation, and dissolution of 0.04 g of residue in 1 mL of water. Fe_3_O_4_ nanoparticles were synthesized by mixing 5 g of the extract with 500 mg of FeCl_3_ in 4 mL of water, heating to 80 °C, and adding 10 mL of 1.5 M Na_2_CO_3_ dropwise. The nanoparticles were magnetically separated and washed three times with water.

### 2.2 Synthesis of Copper Nanoparticles (CuO NPs)

CuO nanoparticles were synthesized by refluxing a mixture of 5 mg of copper nitrate, 50 mL of *M. pubescens* infusion, and 2.0 g of urea at 80 °C for 5 h. The resulting material was washed and centrifuged three times.

### 2.3 Fabrication of TiO_2_-Coated Fe_3_O_4_ Photocatalyst with Immobilized CuO

The Fe_3_O_4_@TiO_2_–CuO photocatalyst was prepared by dispersing 100 mg of Fe_3_O_4_ in a mixture containing 2 mL of titanium­(IV) isopropoxide, 2 g of urea, 20 mL of glacial acetic acid, 20 mL of octanol, and 10 mg of CuO nanoparticles. The suspension was refluxed at 80 °C for 5 h. The solid was magnetically separated, washed three times with distilled water, dried, and calcined at 450 °C for 2 h. Urea acted as a homogeneous precipitating and complexing agent, promoting controlled hydrolysis of titanium precursors and uniform adhesion of the TiO_2_–CuO shell onto the Fe_3_O_4_ core.

For a clearer visualization of the overall procedure, the complete synthesis route is summarized in the flowchart shown below:

### 2.4 Photocatalytic Activity Tests

Photocatalytic experiments were carried out in a 100 mL double-layered borosilicate glass reactor maintained at 20 °C using a thermostatic bath. A suspension containing 80 mg of Fe_3_O_4_@TiO_2_–CuO photocatalyst in 25 mL of aqueous RhB solution (15 ppm) was irradiated with a 100 W visible white LED light source (Kelo A0100, 380–780 nm, 30 V), positioned approximately 10 cm from the reactor to maximize photon flux and enhance light absorption by the photocatalyst.

RhB degradation was monitored by UV–vis spectrophotometry through absorbance measurements at 554 nm. Aliquots were withdrawn at 60 min intervals to determine the remaining RhB concentration. After irradiation, the photocatalyst was magnetically separated using a neodymium magnet, enabling recovery and reuse. Reaction products were analyzed by GC–MS.

## 3 Characterization

A comprehensive set of experimental and theoretical techniques was used to evaluate the structural, chemical, morphological, magnetic, optical, and photocatalytic properties of the Fe_3_O_4_@TiO_2_–CuO composite. XRD patterns were collected on a Bruker D8 ADVANCE (Cu Kα, λ = 1.5406 Å) and analyzed by Rietveld refinement (GSAS-II) using CIFs from COD, Materials Project, and RRUFF to identify phases, quantify weight fractions, and estimate crystallite sizes. Crystallite sizes were also calculated using the Scherrer equation

1 D=Kλ/(βcos⁡θ)

where *D* is the mean crystallite size, *K* the shape factor, λ the X-ray wavelength, β the fwhm, and θ the Bragg angle. XPS (Thermo K-Alpha, Al Kα, *h*ν = 1486.6 eV) provided surface composition and oxidation-state information, with binding energies referenced to C 1s at 285.0 eV. Bulk composition was determined by XRF (Thermo K-Alpha), and magnetic properties were obtained by VSM at room temperature.

SEM, STEM, and EDX analyses were performed using Thermo Scientific Apreo 2 and FEI Tecnai G^2^ F20 microscopes. ICP–OES (Arcos Series) quantified total metal contents and assessed copper leaching after reuse. FTIR spectra (4000–400 cm^–1^) characterized functional groups in the *M. pubescens* extract and supported the green synthesis pathway. UV–Vis spectroscopy was used to determine optical properties and band gap (Tauc method), while GC–MS was employed to monitor RhB degradation products after photocatalysis.

## 4 Results and Discussion

Figure 1 shows the stacked X-ray diffraction (XRD) patterns of the synthesized Fe_3_O_4_@TiO_2_–CuO composite, where the experimental diffraction pattern is compared with reference patterns simulated from crystallographic information files (CIFs). The observed reflections confirm the coexistence of magnetite (Fe_3_O_4_), hematite (Fe_2_O_3_), anatase-TiO_2_, rutile-TiO_2_, and tenorite (CuO).

**Figure 1:**
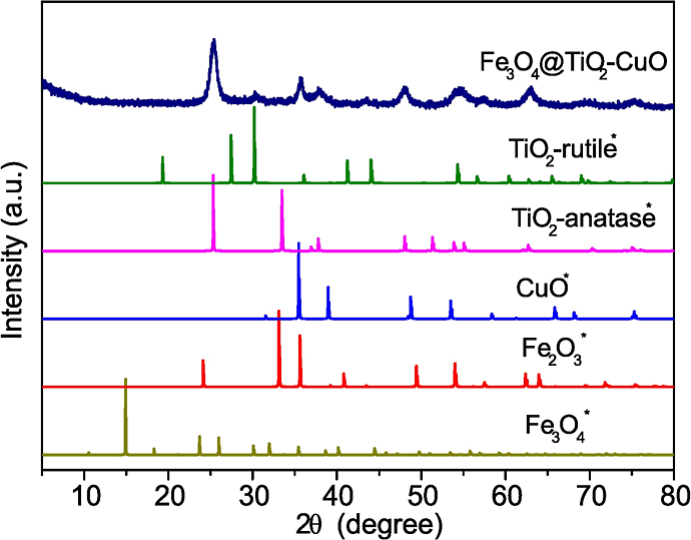
Stacked X-ray diffraction (XRD) patterns of the Fe_3_O_4_@TiO_2_–CuO composite. The experimental diffraction pattern is shown at the top, while reference patterns of Fe_3_O_4_, Fe_2_O_3_, TiO_2_ (anatase and rutile), and CuO were simulated from crystallographic information files (CIFs) and are indicated by an asterisk (*). All diffraction patterns were normalized to their maximum intensity and vertically offset for clarity.

A quantitative structural analysis was further carried out by Rietveld refinement of the experimental XRD pattern using the GSAS-II software, and the corresponding refinement plot is provided in the Supporting Information (Figure S8). The diffraction pattern was successfully modeled considering five crystalline phases: Fe_3_O_4_, Fe_2_O_3_ (hematite), anatase-TiO_2_, rutile-TiO_2_, and CuO. The refined weight fractions were 41.8% for Fe_2_O_3_, 28.4% for Fe_3_O_4_, 20.7% for anatase, 7.4% for rutile, and 1.7% for CuO, indicating that iron oxides dominate the composite, while TiO_2_ is mainly present in the anatase form with a minor rutile contribution.

The refined lattice parameters were consistent with standard crystallographic data. Fe_3_O_4_ crystallized in a cubic structure with *a* = 8.409 Å, while Fe_2_O_3_ adopted a rhombohedral structure with *a* = 8.345 Å. The anatase and rutile polymorphs of TiO_2_ were modeled with tetragonal unit cells, yielding lattice parameters of *a* = 2.691 Å and *c* = 4.748 Å for anatase, and *a* = 4.641 Å and *c* = 2.921 Å for rutile. The CuO phase crystallized in a monoclinic structure with lattice constants of *a* = 6.418 Å, *b* = 3.999 Å, and *c* = 5.198 Å.

Microstrain refinement was reliable only for the Fe_3_O_4_ phase, resulting in a value of 1632 ppm (0.1632%), which suggests significant lattice distortions. These distortions may originate from partial oxidation processes, interfacial stress with adjacent oxide phases, or the nonequilibrium nature of the green synthesis route. Attempts to refine microstrain for Fe_2_O_3_ and CuO led to nonphysical values, indicating that magnetite is the most structurally perturbed phase in the system.

The simultaneous presence of Fe_3_O_4_ and Fe_2_O_3_ indicates partial oxidation of magnetite, likely promoted by the calcination step at 450 °C, which favors the oxidation of Fe^2+^ to Fe^3+^ and the formation of hematite alongside magnetite. Crystallite sizes were refined using an isotropic domain size broadening model, yielding values of 22.7 nm for Fe_3_O_4_, 28.4 nm for Fe_2_O_3_, 17.1 nm for anatase-TiO_2_, and 20.3 nm for rutile-TiO_2_. The CuO phase exhibited an apparent crystallite size of approximately 1.0 nm, which likely reflects either highly disordered domains or uncertainties arising from its low weight fraction and peak overlap with other phases.

Regarding the TiO_2_ component, anatase is clearly the dominant polymorph, as evidenced by its sharper and more intense reflections, particularly the (101) peak at ∼25° (2θ), which displays high intensity and low full width at half-maximum (fwhm), characteristic of well-crystallized anatase domains.^27–29^ In contrast, rutile contributes only weak reflections, such as the (110) plane near ∼27.4° (2θ). This phase distribution is consistent with plant-mediated and low-temperature synthesis routes, which typically favor anatase formation. The coexistence of anatase and rutile, even at low rutile content, may enhance interfacial charge separation, while the predominance of iron oxides ensures the magnetic functionality of the composite, both of which are relevant for its photocatalytic performance.

Figure 2 presents the high-resolution XPS spectra of the main elements identified in the Fe_3_O_4_@TiO_2_–CuO catalyst. The detailed analysis begins with the C 1s spectrum, which exhibits three distinct deconvoluted peaks located at 285.3, 286.2, and 290.4 eV. These peaks represent surface functional groups typically originating from residual plant-derived organic molecules utilized during the synthesis process.

**Figure 2:**
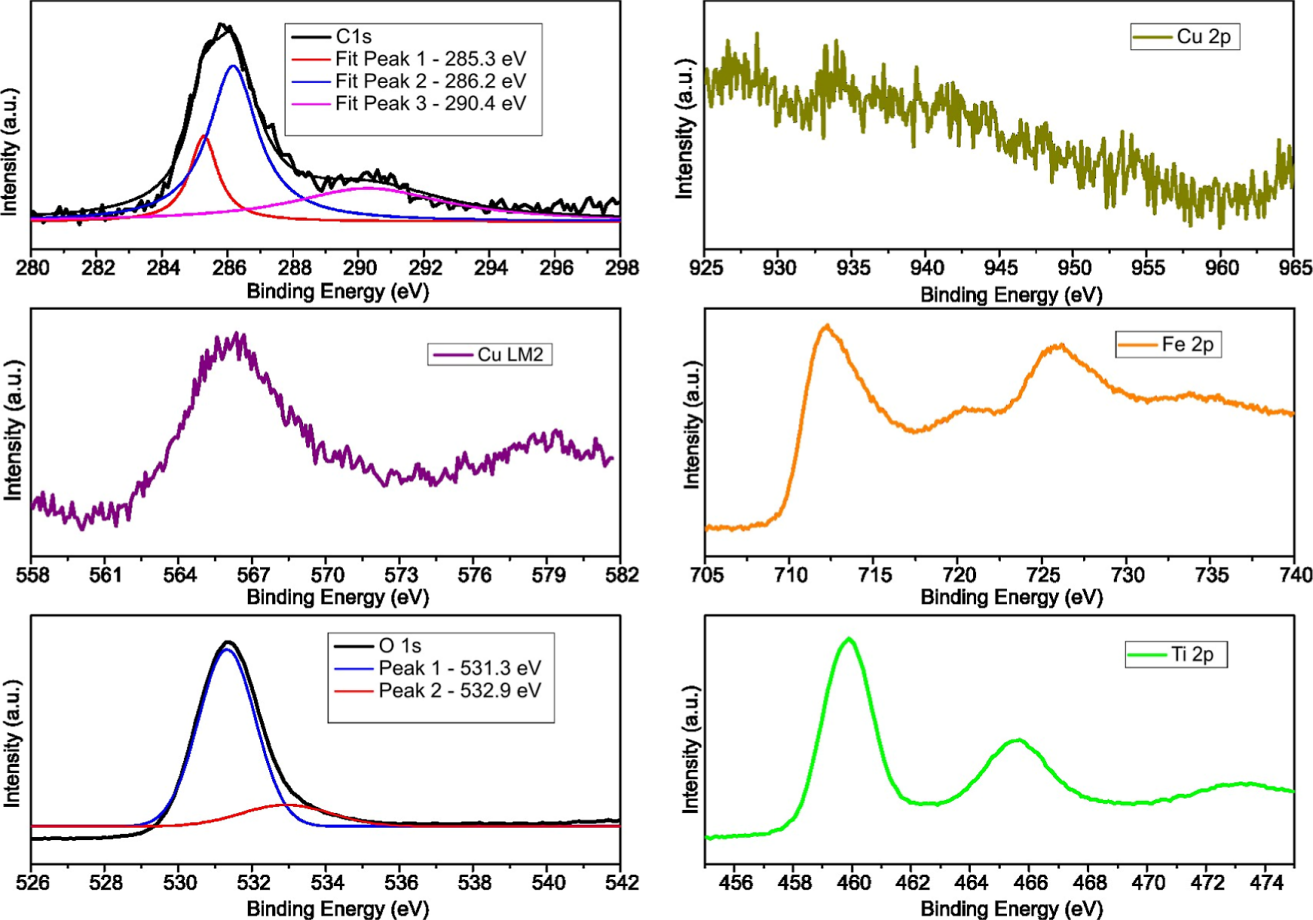
High-resolution XPS spectra for the Fe_3_O_4_@TiO_2_–CuO composite, showing the C 1s, Fe 2p, O 1s, Ti 2p, Cu 2p core levels and the Cu LM2 Auger transition.

The most intense peak at ∼285.3 eV corresponds to C–C and C–H bonds, commonly assigned to adventitious carbon or aliphatic/aromatic carbon groups. Such features are frequently observed in XPS measurements due to hydrocarbon contamination from ambient exposure or residual capping agents derived from plant extracts.^30^ Additionally, this peak commonly serves as a reference for charge calibration.

The second peak, positioned at 286.2 eV, is associated with carbon atoms bonded to oxygen functionalities, including C–O, C–OH, and C–O–C groups.^31^ These functional groups indicate the presence of alcohols, phenols, and ethers, characteristic of polyphenols, lignin derivatives, and saccharides abundant in botanical extracts. The retention of these groups on the nanomaterial surface confirms effective stabilization and capping of the composite via organic moieties during the green synthesis approach.

The third component at 290.4 eV is indicative of highly oxidized carbon species, such as carbonate groups and CO_2_ groups.^32,33^ These functionalities may originate from uronic acids, tannins, or oxidative degradation products of polyphenols present in the plant matrix. The occurrence of these functional groups highlights extensive oxidation on the surface, potentially offering coordination sites for metal ions and enhancing catalytic activity.

The presence of these three carbon-based components indicates that organic residues from the plant-mediated synthesis remain on the Fe_3_O_4_@TiO_2_–CuO nanocomposite surface. These surface functionalities may be beneficial, potentially enhancing dispersion, colloidal stability, and surface reactivity of the composite.

The high-resolution Fe 2p spectrum reveals two clearly defined peaks at approximately 712.2 eV (Fe 2p_3_/_2_) and 725.4 eV (Fe 2p_1_/_2_). These signals indicate the coexistence of Fe­(II) and Fe­(III) oxidation states, typical of iron oxides. Notably, the observed Fe 2p_3_/_2_ peak at 712.2 eV is shifted to higher binding energy compared to typical values (around 710.8 eV) reported for pure magnetite (Fe_3_O_4_).^34^ This shift suggests an interaction between Fe and TiO_2_ within the composite, modifying the local electronic environment around Fe atoms and potentially enhancing charge transfer phenomena. Such interactions at the Fe–TiO_2_ interface may significantly influence catalytic properties or surface reactivity. The presence of satellite features, characteristic of Fe­(III) species,^35^ further confirms iron oxidation and underscores the complex interactions within the nanocomposite structure.

The high-resolution O 1s spectrum of the Fe_3_O_4_@TiO_2_–CuO composite synthesized via plant extracts reveals two distinct deconvoluted peaks centered at approximately 531.0 and 532.0 eV. The peak at 531.0 eV primarily corresponds to lattice oxygen (O^2–^) species bonded to metal cations (Fe, Ti, Cu) within the oxide structure, consistent with Fe–O, Ti–O, and Cu–O bonds.^36^ The second peak at 532.0 eV represents surface-adsorbed oxygen species, including oxygen vacancy signals,^37^ chemisorbed water, or oxygen-containing functionalities derived from residual organic compounds used during synthesis.

The Ti 2p XPS spectrum exhibits two distinct peaks at approximately 458.6 eV (Ti 2p_3/2_) and 464.3 eV (Ti 2p_1/2_). The energy separation of ∼5.7 eV between these peaks aligns with the Ti^4+^ oxidation state characteristic of TiO_2_, confirming the successful formation of titanium dioxide. Importantly, the absence of signals around 457.5 eV indicates negligible presence of reduced Ti^3+^ species, suggesting integrity and stability of the TiO_2_ lattice within the Fe_3_O_4_@TiO_2_ composite.^38,39^

Finally, the surface characterization of the Fe_3_O_4_@TiO_2_–CuO composite was complemented by copper-specific analysis. Despite careful spectral analysis, no distinguishable signal was observed in the Cu 2p region (920–970 eV). This absence of detectable peaks can likely be attributed to the very low copper concentration (approximately 0.3%, as determined by X-ray fluorescence (XRF) analysis), which may fall below the sensitivity limits of conventional XPS measurements. It is important to note that, in preliminary experiments, increasing the CuO content above the optimized value of ∼0.3% resulted in a pronounced decrease in photocatalytic activity. This effect is likely due to excessive surface coverage of TiO_2_ by CuO domains, which can block active sites and hinder light absorption.

Nevertheless, a clear signal was identified in the Cu LMM Auger region (Cu LM2), appearing prominently between 565 and 575 eV. The observed Cu LMM Auger peak is centered near approximately 568–569 eV, indicating copper predominantly in the Cu^2+^ oxidation state (CuO). Such a finding aligns well with the anticipated chemical nature of copper oxide within the Fe_3_O_4_@TiO_2_–CuO composite, confirming the successful incorporation of copper species in their oxidized form at the material’s surface.

To further evaluate the influence of copper oxide incorporation, a comparative analysis was performed between Fe_3_O_4_@TiO_2_–CuO and Fe_3_O_4_@TiO_2_ (without CuO) materials (Supporting Information 1). Notably, the Fe 2p region in the Fe_3_O_4_@TiO_2_ material exhibited similar main peaks at around 710.8 eV (Fe 2p_3/2_) and 724.5 eV (Fe 2p_1/2_); however, the satellite peaks were significantly less intense compared to Fe_3_O_4_@TiO_2_–CuO. This reduced intensity of satellite features indicates a lower relative contribution of Fe­(III) species or diminished electron correlation effects, suggesting a predominant magnetite-like (Fe_3_O_4_) phase.

The C 1s spectrum for Fe_3_O_4_@TiO_2_ showed similar carbon functionalities, with peaks at 285.5 eV (C–C/C–H), 286.7 eV (C–O), and 290.2 eV (O–C=O). Slight peak shifts in the Fe_3_O_4_@TiO_2_–CuO composite (285.3, 286.2, and 290.4 eV) suggest minor alterations to surface chemistry, likely due to copper incorporation. In the O 1s region, the lattice oxygen peak at approximately 531.0 eV remained consistent for both materials. However, the surface-adsorbed oxygen species slightly shifted from 531.9 eV (Fe_3_O_4_@TiO_2_) to 532.0 eV (Fe_3_O_4_@TiO_2_–CuO), reflecting subtle modifications in surface reactivity associated with the addition of copper oxide. Titanium spectra (Ti 2p) showed no notable differences between both composites, indicating the integrity of the TiO_2_ structure upon CuO incorporation.

Additionally, the Fe_3_O_4_@TiO_2_–CuO composite after deactivation in the photodegradation of RhB (Fe_3_O_4_@TiO_2_–CuO-deac) was also analyzed (Supporting Information 2). Notably, the Fe 2p spectrum showed minimal changes, indicating the stability of iron species during catalytic use. However, the O 1s spectrum displayed a slight shift of surface-adsorbed oxygen species to higher binding energy (532.7 eV), suggesting surface oxidation or accumulation of adsorbed species during the photocatalytic process. The C 1s region revealed significant changes with only two peaks at 286.2 eV (C–O) and 292.4 eV (O–C=O), suggesting oxidative degradation or removal of aliphatic/aromatic carbon groups from the surface after prolonged photocatalytic activity. The Cu LMM Auger peak remained consistent around 568–569 eV, confirming the retention of Cu^2+^ (CuO) species postcatalysis.

These comparative results confirm that CuO incorporation primarily influences the local electronic environment around iron and subtly alters surface chemistry without compromising the fundamental TiO_2_ lattice structure.

The optical band gaps of Fe_3_O_4_@TiO_2_, Fe_3_O_4_@TiO_2_–CuO, and the deactivated Fe_3_O_4_@TiO_2_–CuO photocatalyst were estimated using the Tauc method, as shown in Figure 3A–C. The band gap energies were extracted by extrapolating the linear region of the [*F*(*R*)*h*ν]^2^ vs *h*ν plots to the *x*-axis, corresponding to the optical absorption edge.

**Figure 3:**
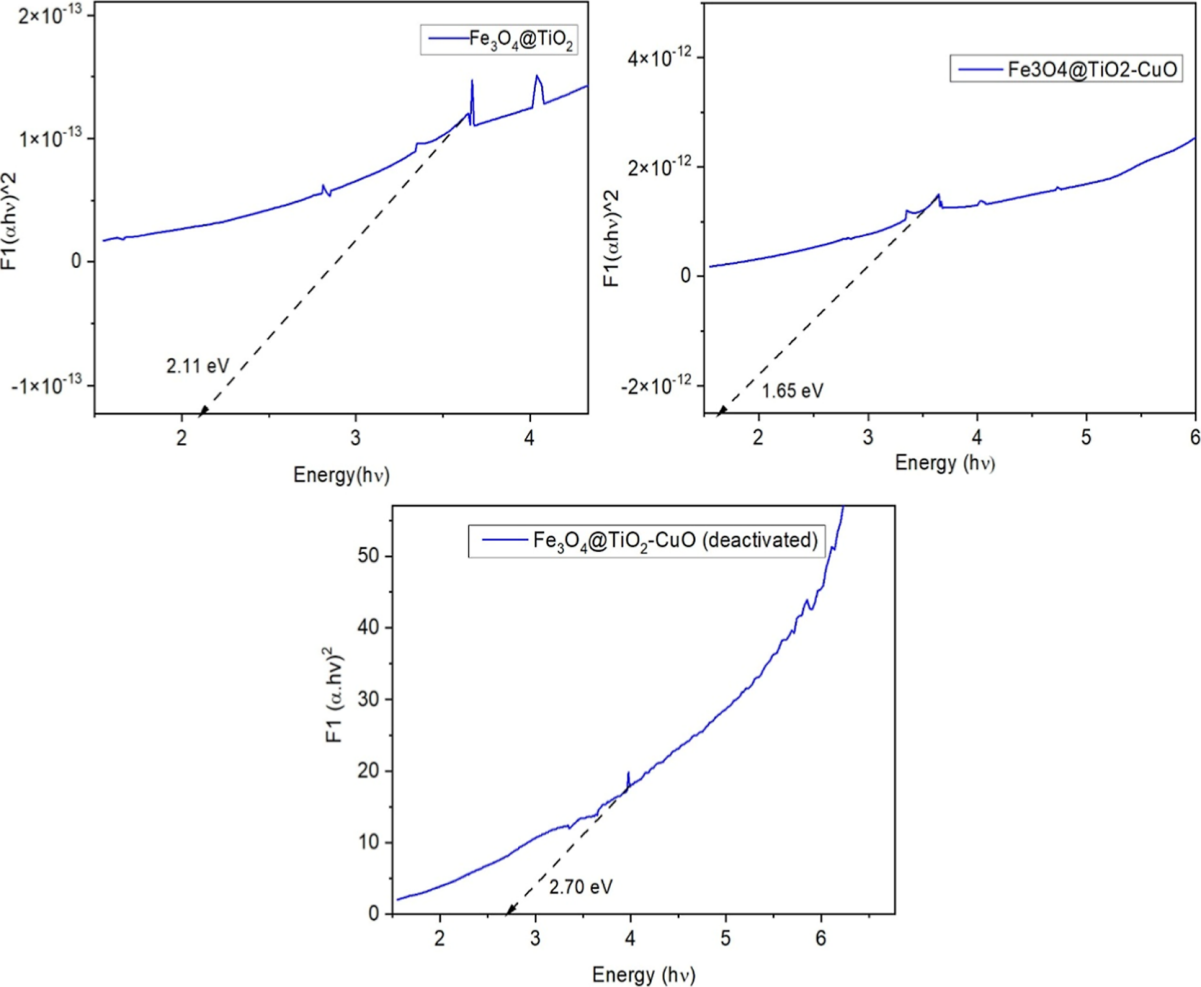
Tauc plots used to estimate the optical band gaps of Fe_3_O_4_@TiO_2_, Fe_3_O_4_@TiO_2_–CuO, and Fe_3_O_4_@TiO_2_–CuO after photocatalytic deactivation.

For the pristine Fe_3_O_4_@TiO_2_ material, the band gap was estimated at 2.11 eV, which already indicates a reduction in the band gap compared to pure TiO_2_ (typically ∼3.2 eV for anatase).^40^ This narrowing is likely due to the incorporation of Fe_3_O_4_, which introduces intermediate energy levels that enhance visible-light absorption.^41^

Upon incorporation of CuO, the composite Fe_3_O_4_@TiO_2_–CuO exhibited a further reduction in band gap to 1.65 eV. This substantial shift can be attributed to the synergistic electronic interaction between Cu­(II) oxide and the existing semiconductor matrix. CuO, being a p-type semiconductor with a narrow band gap (∼1.2–1.7 eV), introduces additional localized states near the valence band, facilitating a red shift in the absorption edge. This enhanced visible-light harvesting capacity is beneficial for photocatalytic applications.

After photocatalytic use, the deactivated Fe_3_O_4_@TiO_2_–CuO sample exhibited a pronounced blue shift, with the band gap increasing to 2.7 eV. This widening indicates surface or structural modifications induced by prolonged irradiation, such as surface oxidation, partial Cu^2+^ leaching, or changes in the electronic environment. The reduction of Cu-related states likely restores a wider band structure, thereby decreasing visible-light absorption and photocatalytic efficiency.

Taken together, these results highlight the effectiveness of CuO incorporation in band gap engineering and photocatalytic enhancement. However, they also point to the need for stability improvements, as the optical properties degrade significantly upon reuse, as reflected in the increased band gap of the deactivated material.

The magnetic behavior of the Fe_3_O_4_@TiO_2_–CuO composite was investigated by vibrating sample magnetometry (VSM) at room temperature. The resulting magnetization curve is shown in Supporting Information S94. The material exhibits a typical ferromagnetic hysteresis loop with well-defined saturation and negligible coercivity, which is further evidenced by the magnified view of the central region included as an inset.

The saturation magnetization (*M*_s_) reached approximately 28.4 emu/g, indicating a strong magnetic response, which can be primarily attributed to the Fe_3_O_4_ core. The incorporation of both TiO_2_ and CuO in the composite structure did not suppress the overall magnetic behavior to a significant extent, suggesting that the magnetic core remained largely intact and magnetically accessible.

The inset highlights the central region of the hysteresis loop, showing a coercivity (*H*_c_) close to zero and negligible remanent magnetization. This is indicative of superparamagnetic-like behavior, which is highly advantageous for practical applications that require rapid magnetic separation and redispersion, such as in photocatalytic or environmental remediation processes. Superparamagnetism ensures that the material does not retain magnetization in the absence of an external magnetic field, thus preventing agglomeration and preserving its colloidal stability in suspension.

These results demonstrate that the Fe_3_O_4_@TiO_2_–CuO composite retains excellent magnetic properties, combining high saturation magnetization with soft magnetic behavior, making it highly suitable for magnetically recoverable catalysts.

The FTIR spectrum of the *M. pubescens* A. St. Hill. (*Sapindaceae*) extract used in the preparation of the photocatalysts is presented in Supporting Information 3. The spectrum exhibits a broad and intense absorption band around 3370 cm^–1^, which is assigned to the stretching vibrations of hydroxyl (O–H) groups commonly found in phenolic compounds and alcohols. This band may also include contributions from N–H stretching modes of amines or amide groups, indicating the possible presence of nitrogen-containing biomolecules such as proteins or alkaloids. In the region between 2920 and 2850 cm^–1^, two distinct peaks are observed, corresponding to the asymmetric and symmetric stretching vibrations of aliphatic C–H bonds from –CH_2_ and –CH_3_ groups.^39^ These signals are indicative of the presence of long-chain hydrocarbons or terpenoids, which are frequently found in plant extracts.

A relatively sharp band observed near 1715 cm^–1^ is attributed to the stretching vibrations of carbonyl (C=O) groups, suggesting the presence of carboxylic acids, esters, or aldehydes.^42^

This signal, together with the one centered around 1625 cm^–1^, which corresponds to C=C stretching in aromatic rings or amide C=O stretching, supports the presence of flavonoid-type compounds and other aromatic constituents. Additionally, the spectrum presents bands around 1450 and 1350 cm^–1^, which are typically assigned to bending vibrations of CH_2_ groups and to C–N stretching of aromatic amines or amino acids.^43^

A strong absorption band between 1000 and 1100 cm^–1^ is attributed to C–O stretching vibrations of alcohols, ethers, and glycosidic linkages,^44,45^ which is consistent with the presence of carbohydrates and polyphenols. Several additional signals below 900 cm^–1^ appear in the fingerprint region and correspond to bending vibrations of aromatic C–H bonds and other skeletal deformations typically associated with complex phytochemical structures.

Together, these spectral features indicate the presence of multiple functional groups, including hydroxyl, carbonyl, aromatic, and aliphatic moieties, revealing a rich phytochemical profile. The abundance of oxygenated and nitrogenated functional groups further suggests that the *M. pubescens* extract contains diverse biomolecules capable of acting as both reducing and stabilizing agents during the synthesis of metal-based photocatalysts.

The FT-IR analysis presented in this work provides clear evidence that plant-derived biomolecules from the *M. pubescens* extract participate in the green synthesis of the Fe_3_O_4_@TiO_2_–CuO nanocomposite. The presence of characteristic absorption bands associated with hydroxyl, carboxyl, and aromatic groups indicates that polyphenols, flavonoids, and organic acids remain at least partially on the nanoparticle surfaces after synthesis. These functional groups are widely reported as key contributors to metal-ion reduction and nanostructure stabilization in phytosynthesis routes. Therefore, the FT-IR data support a mechanism in which these biomolecules act as reducing, chelating, and stabilizing agents during nanoparticle formation and coating.

Figure 4 shows SEM images of the synthesized Fe_3_O_4_@TiO_2_–CuO composite at different magnifications, providing insight into the material’s morphology across multiple length scales. At lower magnification, the composite appears as irregular micrometric aggregates with nonuniform shapes, indicating a strong tendency toward agglomeration. This behavior is commonly observed in magnetic nanocomposites and is primarily associated with magnetic dipole–dipole interactions among Fe_3_O_4_ cores.

**Figure 4:**
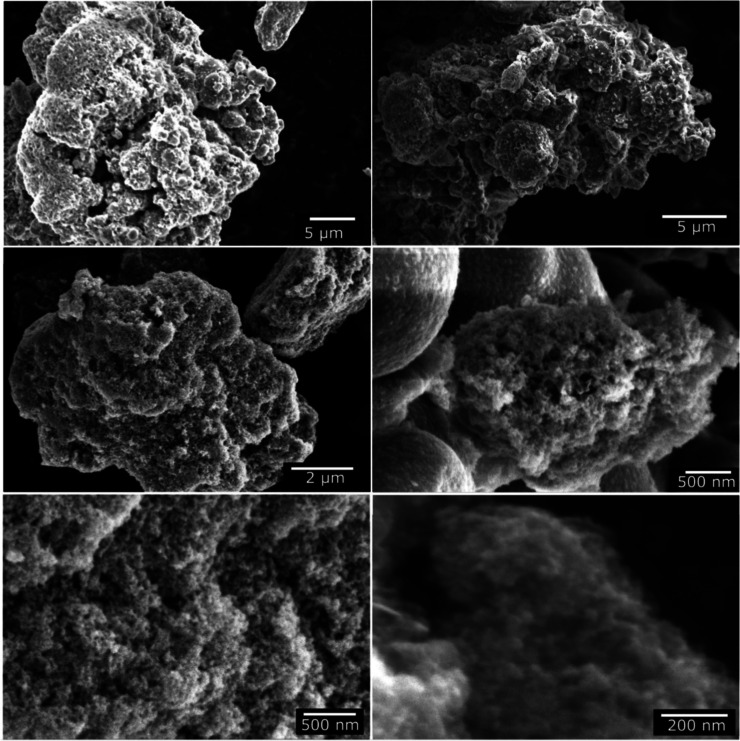
SEM images showing the morphology of the synthesized material at different length scales.

Higher-magnification SEM images reveal that these micrometric clusters are composed of nanosized primary particles, forming a rough and highly textured surface. The aggregates exhibit a heterogeneous morphology with loosely packed domains and pronounced surface irregularities. Such features suggest the presence of interparticle voids and an open architecture within the agglomerates.

Although SEM does not provide direct information on internal porosity, the combination of surface roughness, granular texture, and incomplete packing of the primary particles indicates a porous-like morphology dominated by interparticle spaces. This structural arrangement is advantageous for catalytic applications, as it may enhance mass transport and increase the accessibility of surface-active sites.

Figure 5 presents DF-STEM and HAADF-STEM images together with elemental mapping of the Fe_3_O_4_@TiO_2_–CuO composite. The DF-STEM images reveal irregular agglomerates composed of smaller nanocrystallites. These particles form a loosely packed architecture with noticeable interparticle voids, which is characteristic of an intergranular porous structure. The circular features seen in the bright-field images originate from the carbon/Formvar TEM support grid containing Au markers and should not be interpreted as intrinsic particles of the composite.

**Figure 5:**
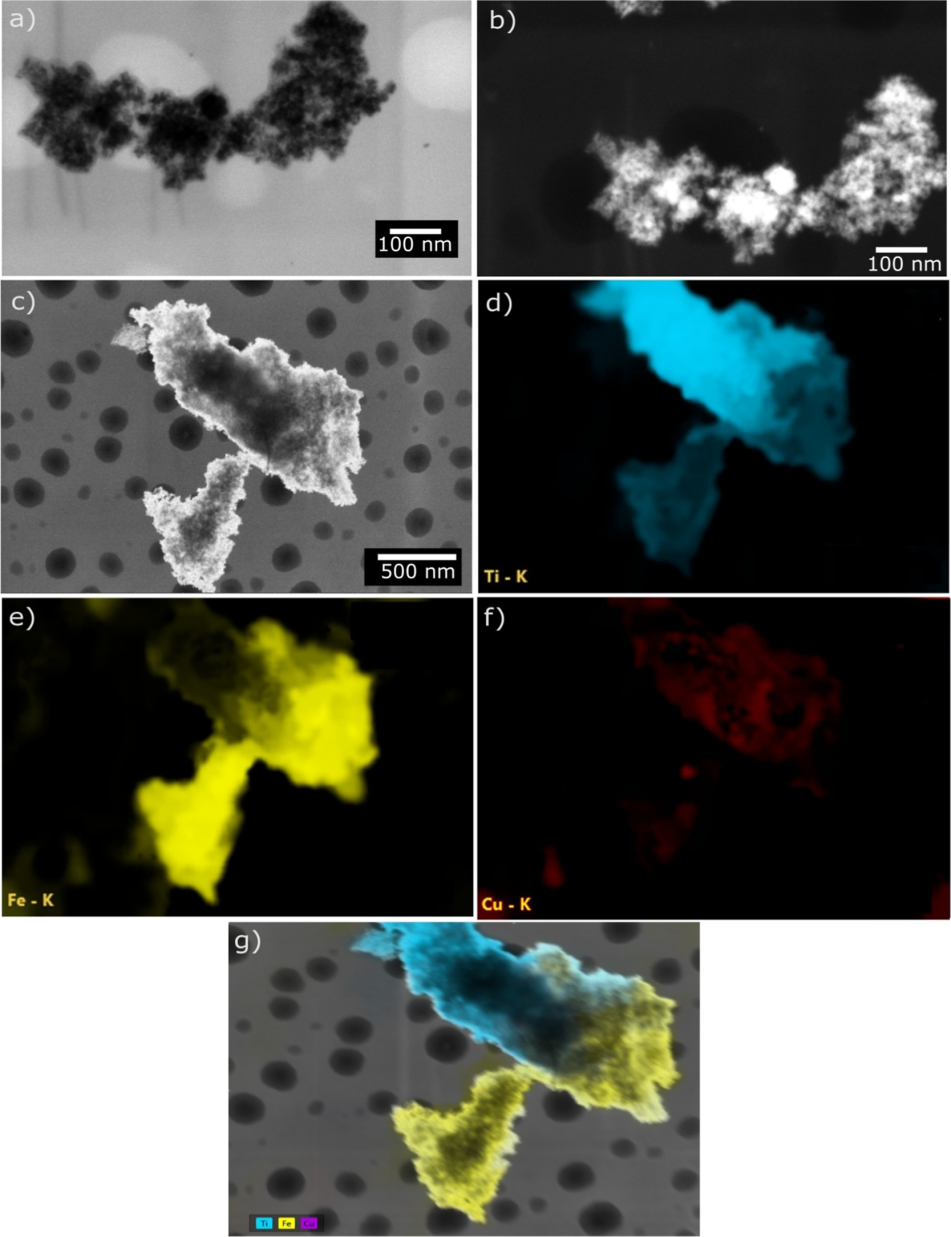
Presents the STEM characterization of the Fe_3_O_4_@TiO_2_–CuO composite, including DF-STEM images at different magnifications (a,b), a corresponding HAADF-STEM image (c), the EDS elemental maps for Ti, Fe, and Cu (d–f), and the combined overlay of these maps with the STEM micrograph (g).

The HAADF-STEM images show pronounced contrast variations within the aggregates, reflecting local differences in average atomic number. This contrast confirms the heterogeneous nature of the multicomponent material. Based on the synthetic route—where CuO nanoparticles were preformed and subsequently introduced during the sol–gel growth of TiO_2_—the elemental distribution observed by HAADF-STEM/EDS mapping is consistent with a dispersed and interfacial CuO phase. Copper appears broadly distributed across the aggregates, without evidence of a continuous shell or well-defined CuO domains. This distribution suggests that CuO nanoparticles are immobilized and partially embedded within the TiO_2_ matrix or positioned at Fe_3_O_4_/TiO_2_ interfaces, where they act primarily as electronically active sites rather than as structural components.

In contrast, Fe and Ti present a less uniform and partially segregated distribution. This observation indicates the coexistence of Fe_3_O_4_- and TiO_2_-rich domains, instead of a fully intermixed or well-defined core–shell architecture.

Quantitative EDS analysis yields atomic percentages of approximately 52.3 at. % Ti, 42.1 at. % Fe, and 5.7 at. % Cu (corresponding to 48.0 wt % Ti, 45.1 wt % Fe, and 6.9 wt % Cu). The partial spatial heterogeneity observed for Fe and Ti can be rationalized by the sequential synthesis route, in which Fe_3_O_4_ nanoparticles were first formed and subsequently coated with TiO_2_, leading to locally varying TiO_2_ coverage across the magnetic cores. The combined STEM–EDS analysis evidence intimate interfacial contact among Fe_3_O_4_, TiO_2_, and CuO phases.

Photocatalytic degradation tests were performed in a 100 mL double-layer borosilicate reactor maintained at 20 °C. A suspension containing 80 mg of Fe_3_O_4_@TiO_2_–CuO and 25 mL of RhB solution (15 ppm) was stirred in the dark for 30 min to reach adsorption equilibrium, followed by visible-light irradiation using a 100 W LED source positioned 10 cm from the reactor. After irradiation, the catalyst was magnetically recovered, washed, and reused for up to four additional cycles.

As shown in Figure 6, the photocatalyst achieved 75% RhB degradation after 5 h of visible-light exposure, reducing the dye absorbance from 0.9114 to 0.2301. This performance demonstrates the material’s potential for dye removal and wastewater treatment. The degradation followed pseudo-first-order kinetics, with an apparent rate constant of 0.00549 min^–1^ (*R* ^2^ = 0.875) and a calculated half-life of approximately 126 min.

**Figure 6:**
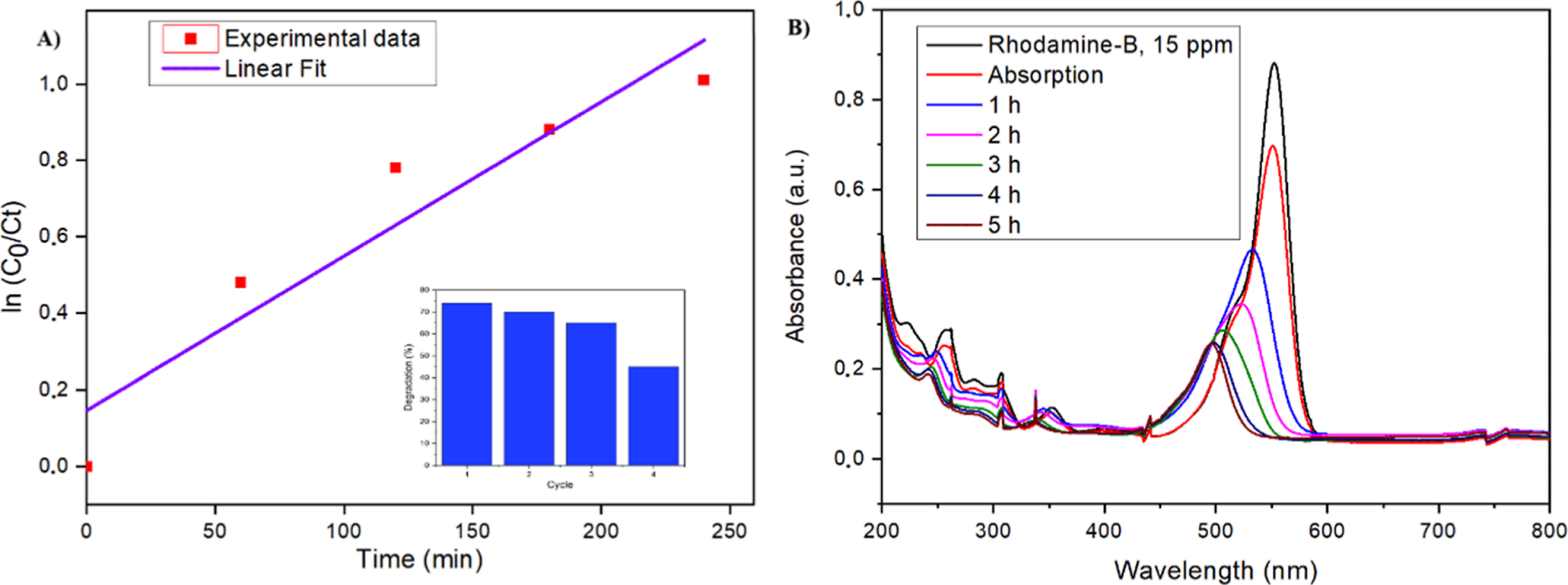
(A) Pseudo-first-order kinetic analysis of rhodamine B degradation under photocatalytic conditions; the inset shows the degradation efficiency over consecutive reuse cycles. (B) Time-dependent UV–Vis absorption spectra of Rhodamine B (15 ppm) during photocatalytic treatment following a 30 min dark adsorption step.

Furthermore, hourly UV–vis spectroscopic analyses conducted throughout the irradiation period (1 to 5 h) revealed a steady decline in absorbance, directly correlating to the progressive reduction of dye concentration. The initial absorbance of RhB (15 ppm) measured without catalyst or light was used as the control. A marked decrease after 30 min in the dark indicated strong dye adsorption, followed by progressive degradation under illumination. The degradation rate leveled off after ∼5 h, likely due to active-site saturation or electron–hole recombination. Such a plateau in degradation efficiency has also been reported in earlier studies,^46–48^ where it was mainly associated with the recombination of photogenerated electron–hole pairs limiting the photocatalytic activity over time.

The catalyst maintained its photocatalytic activity for up to four reuse cycles, although efficiency declined by approximately 30% by the final cycle. Specifically, the degradation efficiency decreased from 73.9% (first reuse, absorbance 0.238) to 45.3% (fourth reuse, absorbance 0.414), with the sharpest decline (20.5%) observed between the third (65.8%) and fourth reuse cycles. The decline in activity likely results from structural modifications of the TiO_2_ phase, partial blockage of active sites by degradation intermediates, or morphological changes impacting the catalyst surface. Additional cycles were not performed, since the significant activity loss indicated that the catalyst’s performance had fallen below practical applicability for further reuse.

Comparative photocatalytic tests revealed that Fe_3_O_4_@TiO_2_–CuO composites exhibit significantly enhanced performance compared to Fe_3_O_4_@TiO_2_, achieving a 75% degradation efficiency versus 37%, which can be ascribed to the formation of an interfacial type-II heterojunction between TiO_2_ and CuO. In this configuration, the conduction band (CB) edge of TiO_2_ is higher than that of CuO, while the valence band (VB) of CuO is higher than that of TiO_2_. Upon visible-light irradiation, photogenerated electrons in the CB of CuO transfer to the CB of TiO_2_, while holes migrate from the VB of TiO_2_ to the VB of CuO. This spatial separation of charge carriers effectively reduces recombination rates and enhances charge utilization in redox reactions at the surface.

Additionally, Cu doping within the TiO_2_ lattice introduces localized states within the band gap, which not only narrows the band gap energy but also promotes visible-light absorption. These modifications contribute synergistically to an improved generation and lifetime of reactive oxygen species (ROS), thereby enhancing photocatalytic degradation of organic pollutants.^49^

Similar electronic structure modifications have been reported for other doped wide-band gap oxides. Computational studies on Zn-substituted β-Ga_2_O_3_ have demonstrated that substitutional dopants introduce localized electronic states within the band gap, altering magnetic and electronic interactions and enabling sub-band gap optical transitions.^50^ In addition, recent combined experimental and computational investigations on Cu-doped anatase TiO_2_ have shown that Cu-induced electronic states and Cu–O–Ti interfacial structures play a central role in visible-light activation and reactive oxygen species generation, particularly superoxide (O_2_^•–^) and hydroxyl (^•^OH) radicals. These findings provide broader mechanistic support for the band gap narrowing and visible-light-driven photocatalytic enhancement observed in the present Fe_3_O_4_@TiO_2_–CuO system.^51^

A notable blue shift observed in the UV–vis spectra during photocatalytic degradation indicates the formation of intermediate degradation products that have shorter wavelengths than the original dye. This phenomenon is generally attributed to the successive removal of chromophoric groups or conjugated double bonds in the dye structure, leading to the formation of simpler, less conjugated molecules with higher band gap energies and shorter wavelength absorptions. It also suggests that the breakdown of the RhB structure occurs through gradual deethylation,^52^ leading to the formation of intermediate products with reduced conjugation.

Although the blue shift itself primarily results from structural changes in the dye molecules due to photodegradation, the use of visible light indirectly influences this process. Specifically, visible light activation enhances the catalyst’s generation of reactive oxygen species, promoting efficient and selective breakdown of conjugated structures^53^ in RhB, thereby accelerating the formation of less conjugated intermediates that produce the observed spectral shift.

The photodegradation of RhB under light irradiation was monitored by gas chromatography–mass spectrometry (GC–MS) at two distinct reaction times: 40 and 300 min (Supporting Information 4). The analysis revealed substantial temporal evolution in the composition of intermediate and final products, evidencing the progression of the photocatalytic breakdown. As shown in Figure S7, the GC–MS profile displays several intermediate fragments, with a signal at *m*/*z* = 282 assigned to a partially deethylated derivative of rhodamine B. This result indicates that the photodegradation proceeds predominantly through an oxidative N-deethylation mechanism promoted by hydroxyl radicals, leading to the successive removal of ethyl groups and gradual cleavage of the chromophoric structure.

After 40 min of photocatalytic treatment using the Fe_3_O_4_@TiO_2_–CuO nanocomposite under visible light, GC–MS analysis revealed a set of intermediate products indicative of advanced degradation of RhB. This observation is consistent with earlier studies reporting that RhB degradation begins rapidly under photocatalytic conditions through a sequence of N-deethylation and ring-opening reactions.^54,55^

The detected products at this early stage include methoxy-phenyl oximes, triazole and pyrrole derivatives, esters, and polyaromatic aldehydes—molecules that reflect specific breakdown pathways of RhB’s xanthene and amino substituents. These findings suggest that photocatalytic attack initiates primarily through oxidative N-deethylation of the diethylamino groups, which are known to be particularly vulnerable to hole- and hydroxyl radical-mediated attack.^56^

This is followed by cleavage of the chromophoric system, which generates colorless but chemically active fragments, such as substituted benzenes, heterocycles, and intermediate carboxylic acids. The formation of 1,2-benzenedicarboxylic acid esters, in particular, may arise from partial mineralization or interaction with residual organic species in solution. This mechanistic interpretation aligns with established pathways where RhB degradation proceeds via sequential dealkylation, deamination, decarboxylation, and aromatic ring-opening, eventually yielding low-molecular-weight acids and alcohols.

From a toxicological perspective, some degradation intermediates, particularly aromatic aldehydes and phthalates, may retain biological activity despite the apparent discoloration of the dye. This reflects a common challenge in photocatalysis, where structurally modified products can still pose environmental risks. Nevertheless, in silico and in vitro studies generally indicate that these intermediates are less persistent and less toxic than the parent compound.

After 300 min of visible-light exposure, the chromatographic profile shifts considerably. A large number of oxidized degradation products appear, including lidocaine, thiazole derivatives, and various aliphatic esters and acids. These low-molecular-weight, oxygen-rich molecules reflect advanced oxidative breakdown and are consistent with literature reports that describe the late-stage mineralization of RhB as proceeding through the formation of small organic acids—including, in many studies, formic, oxalic, and acetic acids, prior to complete mineralization.^57^

The presence of lipid-like molecules, such as 13-docosenamide, may indicate secondary reactions or contributions from the matrix, but overall the data demonstrate progressive simplification of molecular structures through oxidative scission.

The use of a ternary Fe_3_O_4_@TiO_2_–CuO system appears critical to this performance. The combination of TiO_2_ with narrow-band gap CuO extends absorption into the visible-light region, while the inclusion of Fe_3_O_4_ provides magnetic recoverability and may enhance electron–hole separation by acting as an electron sink. These synergistic effects support the observed catalytic efficiency over time, with significant transformation of RhB occurring in the visible light range. This is particularly relevant for real-world wastewater applications, where solar light and recyclability are essential operational criteria.

Interestingly, a noticeable difference was observed after the immobilization of the final CuO phase onto Fe_3_O_4_@ TiO_2_, depending on whether urea was used during synthesis. The urea-free sample produced a visibly darker solution after magnetic separation, suggesting increased leaching of iron species. To investigate this behavior, ICP analysis was performed on both solutions to quantify Fe, Ti, and Cu concentrations. The results revealed a substantially higher Fe concentration in the absence of urea (∼102 ppm) compared to the sample synthesized with urea (∼54 ppm), indicating that urea plays a stabilizing role by minimizing the dissolution of nonmagnetic Fe^3+^ species. In contrast, titanium levels remained nearly identical between the two conditions (0.194% with urea vs 0.187% without), suggesting that TiO_2_ deposition is robust and not significantly influenced by the presence of urea. Copper was undetectable in both cases, confirming effective incorporation and retention of CuO within the magnetically separated composite. These findings underscore the importance of integrating not only photoactive components—such as plant-extract-mediated Fe_3_O_4_, TiO_2_, and CuO, but also chemical modulators like urea, which contribute to the structural stability and environmental resilience of the photocatalyst. The synergistic combination of a green synthetic route with controlled sol–gel processing demonstrates a promising strategy for the development of robust, magnetically recoverable photocatalytic materials for real-world applications.

When the photocatalytic efficiency of the biosynthesized Fe_3_O_4_@TiO_2_–CuO composite is compared to that of other catalytic systems reported in the literature (Table S1), it becomes evident that its performance is comparable to that of similar catalysts synthesized via conventional chemical routes. These findings highlight green, urea-assisted biosynthesis as a viable and competitive route for magnetically separable photocatalysts in environmental applications.

Finally, combining the GC–MS and Tauc results, we propose a comprehensive photocatalytic mechanism for the degradation of rhodamine B under visible-light irradiation (Figure 7).

**Figure 7:**
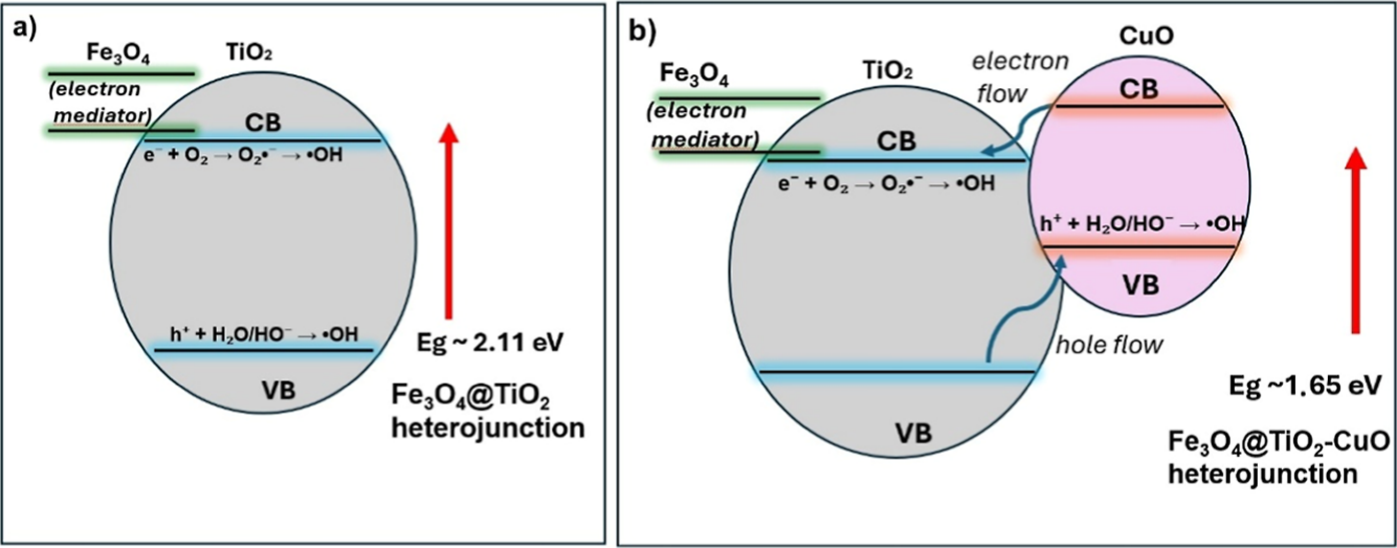
Proposed visible-light photocatalytic mechanism for rhodamine B degradation over (a) Fe_3_O_4_@TiO_2_ and (b) Fe_3_O_4_@TiO_2_–CuO heterojunctions.

Figure 7a illustrates the band structure of the Fe_3_O_4_@TiO_2_ composite, which exhibits an optical band gap of approximately 2.11 eV as obtained from the Tauc analysis. In this system, photogenerated electrons in the TiO_2_ conduction band can reduce dissolved O_2_ to superoxide radicals (O_2_^•–^), while holes in the valence band oxidize H_2_O or HO^–^ to generate hydroxyl radicals (^•^OH). Both ROS contribute to RhB degradation, but the overall visible-light response remains moderate due to the relatively wide band gap.

Upon CuO incorporation, as shown in Figure 7b, the optical band gap decreases to 1.65 eV, improving visible-light absorption. A type-II heterojunction is established between TiO_2_ and CuO, in which photoexcited electrons in the CuO conduction band migrate to TiO_2_ and are subsequently transferred to the Fe_3_O_4_ core acting as an electron mediator. Concurrently, holes move in the opposite direction, from the TiO_2_ valence band to the CuO valence band. This bidirectional charge migration effectively suppresses electron–hole recombination, prolongs carrier lifetime, and enhances the formation of reactive oxygen species. Electrons accumulated in the TiO_2_/Fe_3_O_4_ conduction region reduce O_2_ to O_2_^•–^ and ^•^OH, whereas holes in the CuO valence band oxidize H_2_O/HO^–^ to produce additional ^•^OH radicals. These radicals, along with direct oxidation by valence-band holes (h^+^), drive both radical and nonradical degradation pathways.

Based on the GC–MS analysis (Figure S6 and Table S2), the degradation pathway of Rhodamine B involves a stepwise N-de-ethylation process (*m*/*z* 326 → 282), followed by chromophore cleavage and the formation of low-molecular-weight oxidized fragments (*m*/*z* < 200), which is consistent with the progressive breakdown of the dye into smaller molecular species.

The synergistic effects of (i) CuO incorporation, which narrows the band gap and improves visible-light absorption, (ii) Fe_3_O_4_ acting as an electron mediator that enhances charge separation and magnetic recovery, and (iii) the efficient generation of radical (^•^OH, O_2_^•–^) and nonradical (h^+^) oxidative species account for the superior photocatalytic performance and recyclability of the Fe_3_O_4_@TiO_2_–CuO composite.

## 5 Conclusion

In this work, a multifunctional Fe_3_O_4_@TiO_2_–CuO nanocomposite was successfully synthesized through a green, urea-assisted sol–gel route integrating *M. pubescens* extract as a natural reducing and stabilizing agent. Structural, morphological, and spectroscopic analyses confirmed the formation of a magnetically recoverable composite with a CuO–TiO_2_ type-II heterojunction, whose band gap narrowing and interfacial charge separation enhanced the visible-light photocatalytic degradation of RhB. The material achieved ∼75% dye removal after 5 h of irradiation and generated progressively oxidized intermediates, demonstrating effective photo-oxidation capability.

These results highlight the potential of bioassisted, low-cost, and scalable synthesis strategies for producing efficient magnetic photocatalysts suitable for wastewater treatment and related environmental applications. Future work exploring the photocatalytic response under different illumination conditions may provide further insight into the light-dependent mechanisms governing the activity of this composite.

## Supplementary Material



## Notes

High-resolution XPS spectra; FTIR spectrum of plant extract; GC–MS chromatograms and mechanistic evidence of N-de-ethylation; comparative photocatalytic performance table; additional TEM images; photocatalytic reactor schematic; Rietveld refinement plot; and room-temperature VSM curve (PDF)

## ∥

Department of Chemistry, Federal University of São Carlos, São Carlos, 13565-905, Brazil
