# Supplementary material for: A Green Sol–Gel Route to Fe3O4@TiO2–CuO Photocatalysts with Structural Stability, Visible–Light Activity, and Magnetic Recoverability
Source: ACS Omega. 2026 Mar 4;11(10):15942–55. doi: 10.1021/acsomega.5c09939 (PMC13000648; doi:10.1021/acsomega.5c09939)
Supplement: Supplementary file 1 [file ao5c09939_si_001.pdf]

A Green Sol–Gel Route to Fe_3O_4@TiO_2–CuO Photocatalysts with Structural Stability, Visible-Light Activity, and Magnetic Recoverability

Gabriel Bardella Stelzer[1], Isabella C. Prescilio[1§], Leonardo G. Vasconcelos[2], Andris F. Bakuzis[3], Marcos J. Jacinto[1*]

[1] Nano Materials and Catalysis Laboratory, Institute of Chemistry, Federal University of Mato Grosso, Cuiabá, 78060-900, Brazil.
[2] Laboratory of Research in Natural Products Chemistry and New Synthetic Methodologies in Organic Chemistry, Institute of Chemistry, Federal University of Mato Grosso, Cuiabá, 78060-900, Brazil.
[3] Institute of Physics, Federal University of Goiás, Goiânia, 74690-900, Brazil.

[§] Present address: Department of Chemistry, Federal University of São Carlos, São Carlos, 13565-905, Brazil.

- Corresponding author: marcos.jacinto@ufmt.br

SUPPLEMENTARY MATERIAL

Figure S1. High-resolution XPS spectra of the synthesized $Fe_3O_4@TiO_2$ material: (a) C 1s region showing deconvoluted peaks (b) Fe 2p region displaying characteristic peaks of iron oxides (c) O 1s region with two components (d) Ti 2p region confirming the presence of Ti^{4+}.

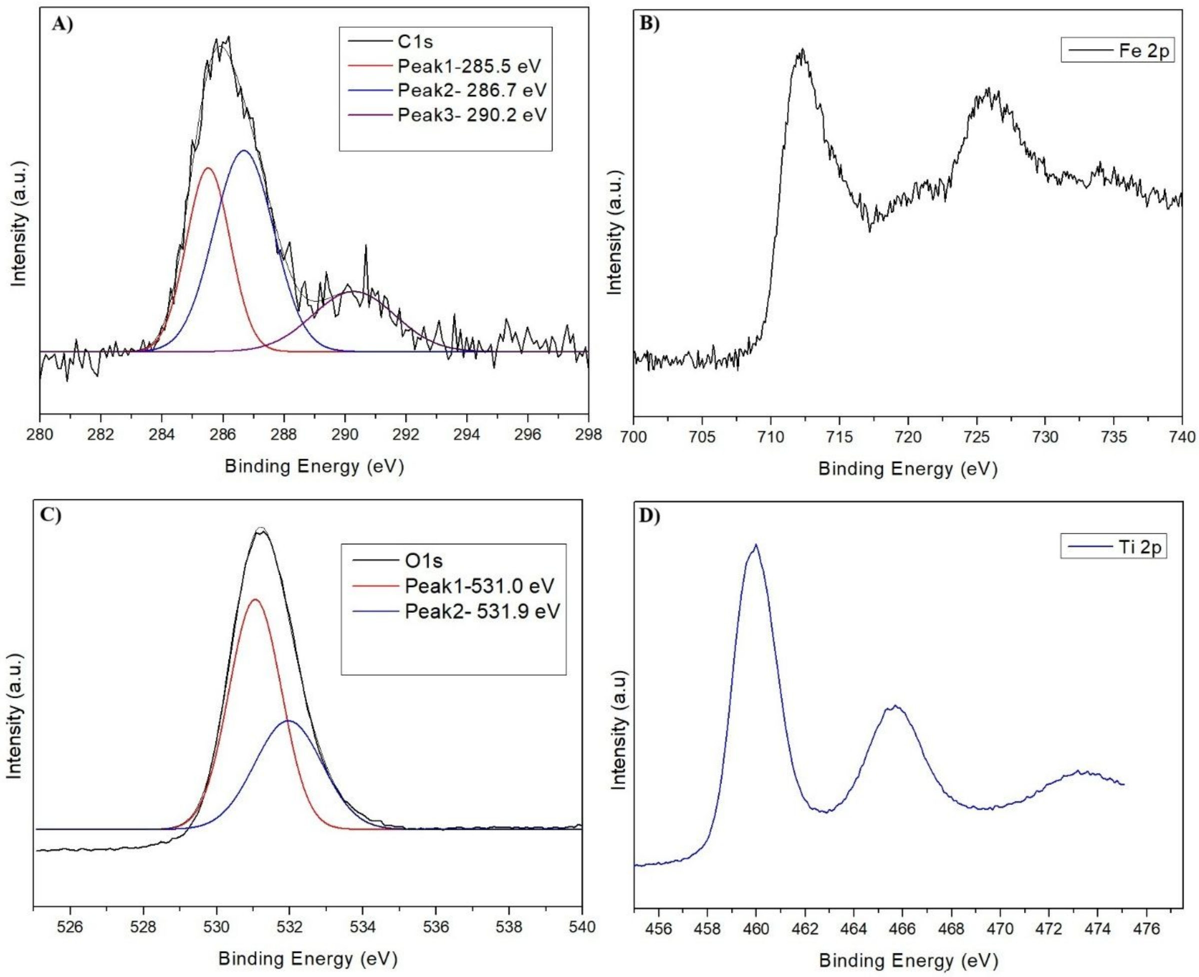

Figure S2. High-resolution XPS spectra of the deactivated Fe_3O_4@TiO_2–Cuo material: (A) C 1s spectrum. (B) Fe 2p region indicating the presence of iron oxide species. (C) O 1s spectrum showing two components. (D) Ti 2p region c (E) Cu 2p region with low-intensity signal in a noisy background. (F) Cu LM_2 Auger peak supporting the presence of copper on the surface.

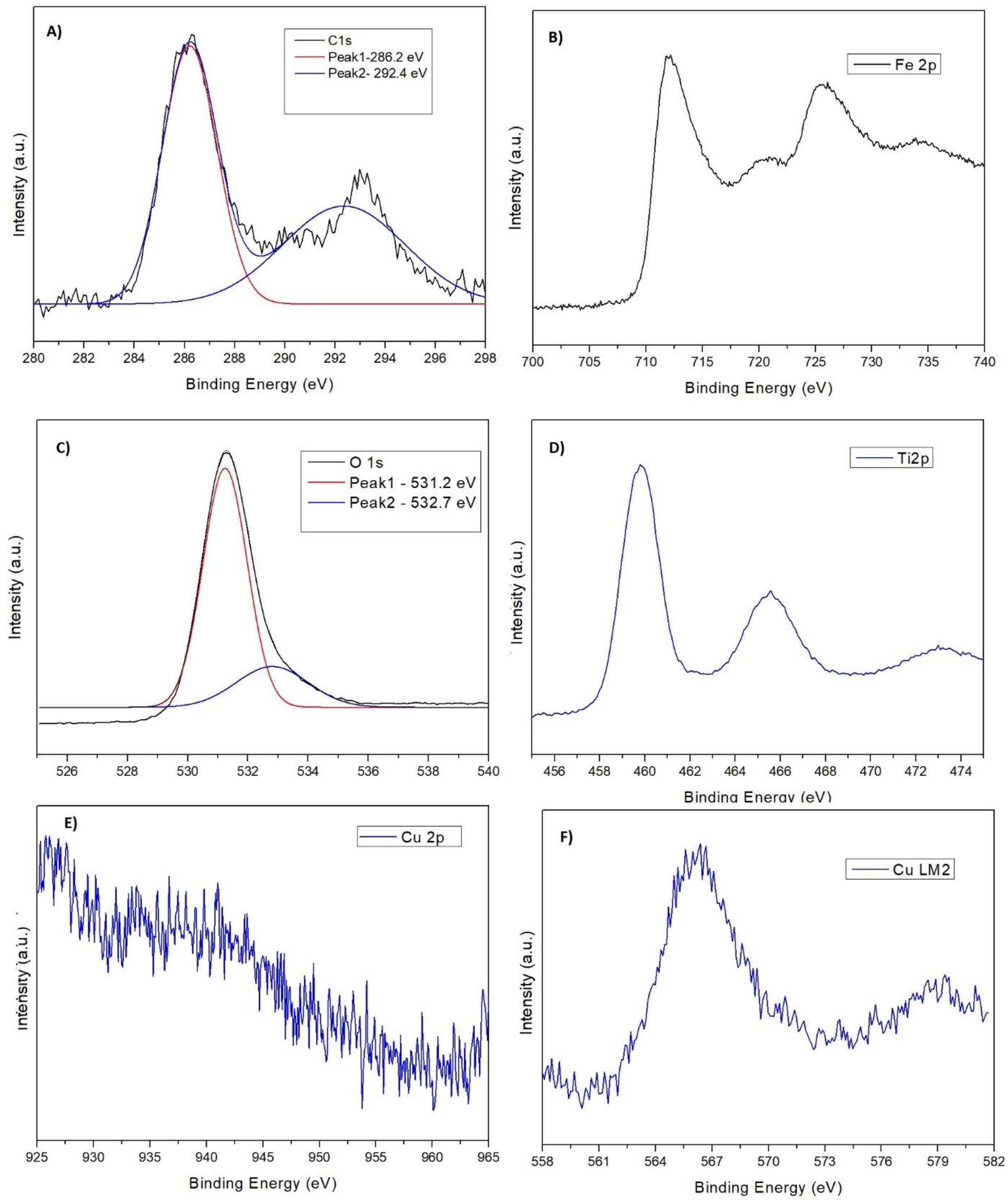

Figure S3. FTIR spectrum of *Magonia pubescens* extract.

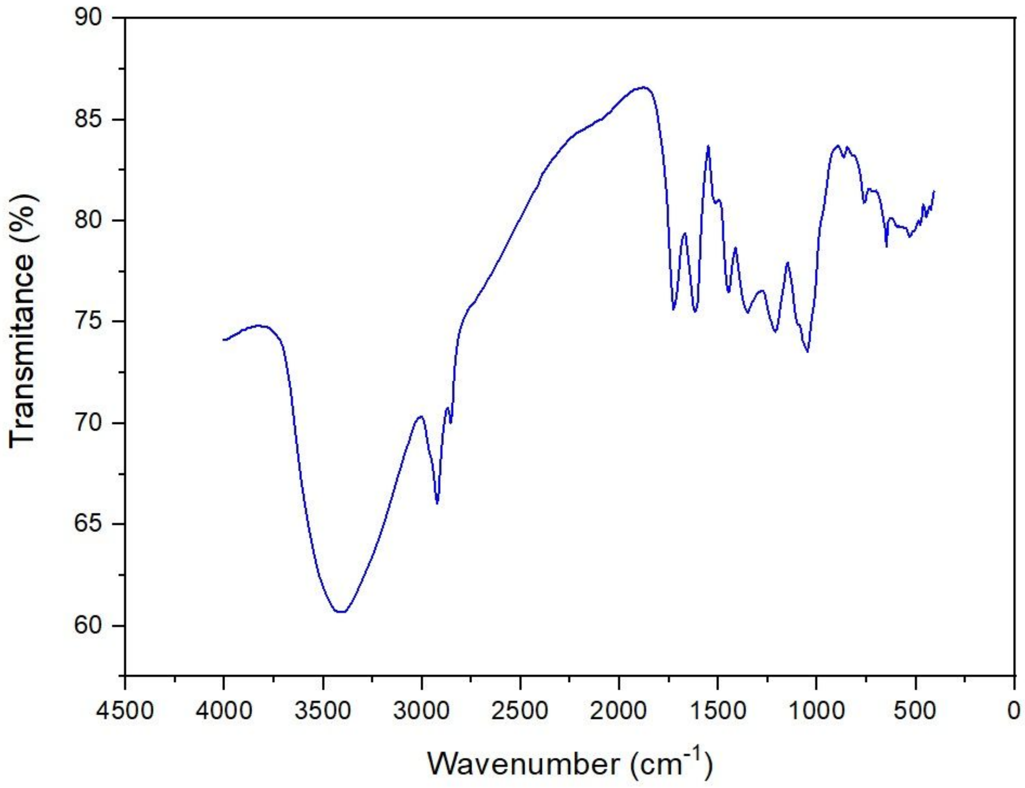

Figure S4. GC-MS chromatograms of the degradation products of Rhodamine B under visible-light irradiation using Fe_3O_4@TiO_2–CuO photocatalyst: (A) After 40 minutes of irradiation, showing several intermediate compounds with lower intensity and complexity; (B) After 300 minutes, revealing a broader range of degradation products.

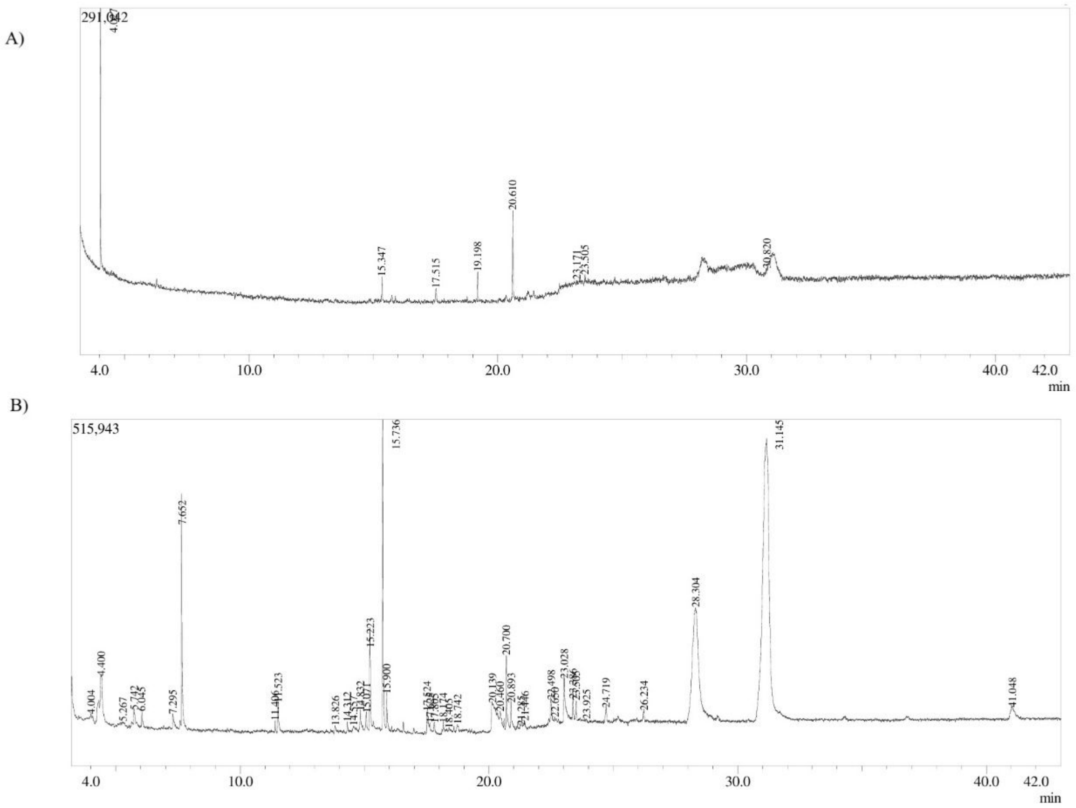

Table S1. Photocatalytic Efficiency comparison of $Fe_3O_4@TiO_2$-CuO with other Reported Catalytic Systems.

**Catalyst**	**Substrate**	**Degradation Efficiency (%)**	**Reation time (h)**	**Recycling (cyles)**	**References**
$Fe_3O_4@TiO_2$-CuO	Rodamine-B	74.75%	5 h	5	This work
$Fe_3O_4/SiO_2/TiO_2$/CuO	Methylene Blue	86%	1.5	5	1
TiO_2-Cu 2%	Methyl orange	55.5%	5	-	2
$Fe_3O_4@TiO_2$@CuO	Methylene Blue	65%	-	-	3
Fe_3O_4/TiO_2/CuO	Azul de Metileno (MB)	100%	0.3 (in the presence of H2O2)	6	4
Fe_3O_4/TiO_2	Crystal violet	86%	3	5	5

Fe_3O_4/TiO_2	2,4-DCP	50%	2	5	6
TiO_2/Cu	Dimethyl formamide	65,6%	3	6	7
Fe_3O_4–TiO_2–SiO_2	Methylene Blue	98%	2	3	8
Fe_3O_4@C–TiO_2–Ag	Methylene Blue	97%	0.17	-	9
Fe_3O_4@SiO_2@TiO_2@Ho	Rhodamine-B	92%	2	7	10

Figure S5. Additional TEM images of the material Fe_3O_4@TiO_2-CuO

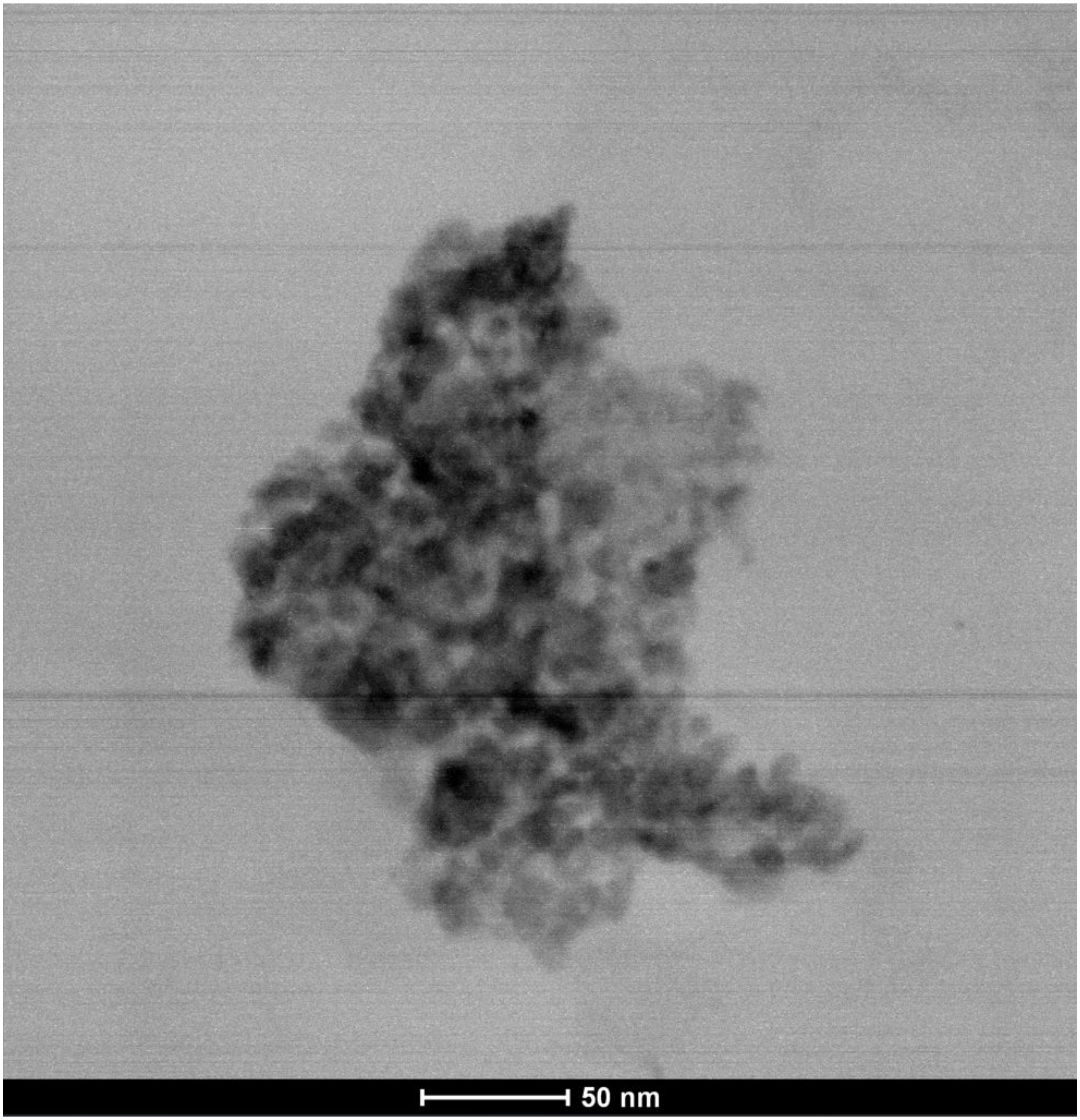
50 nm

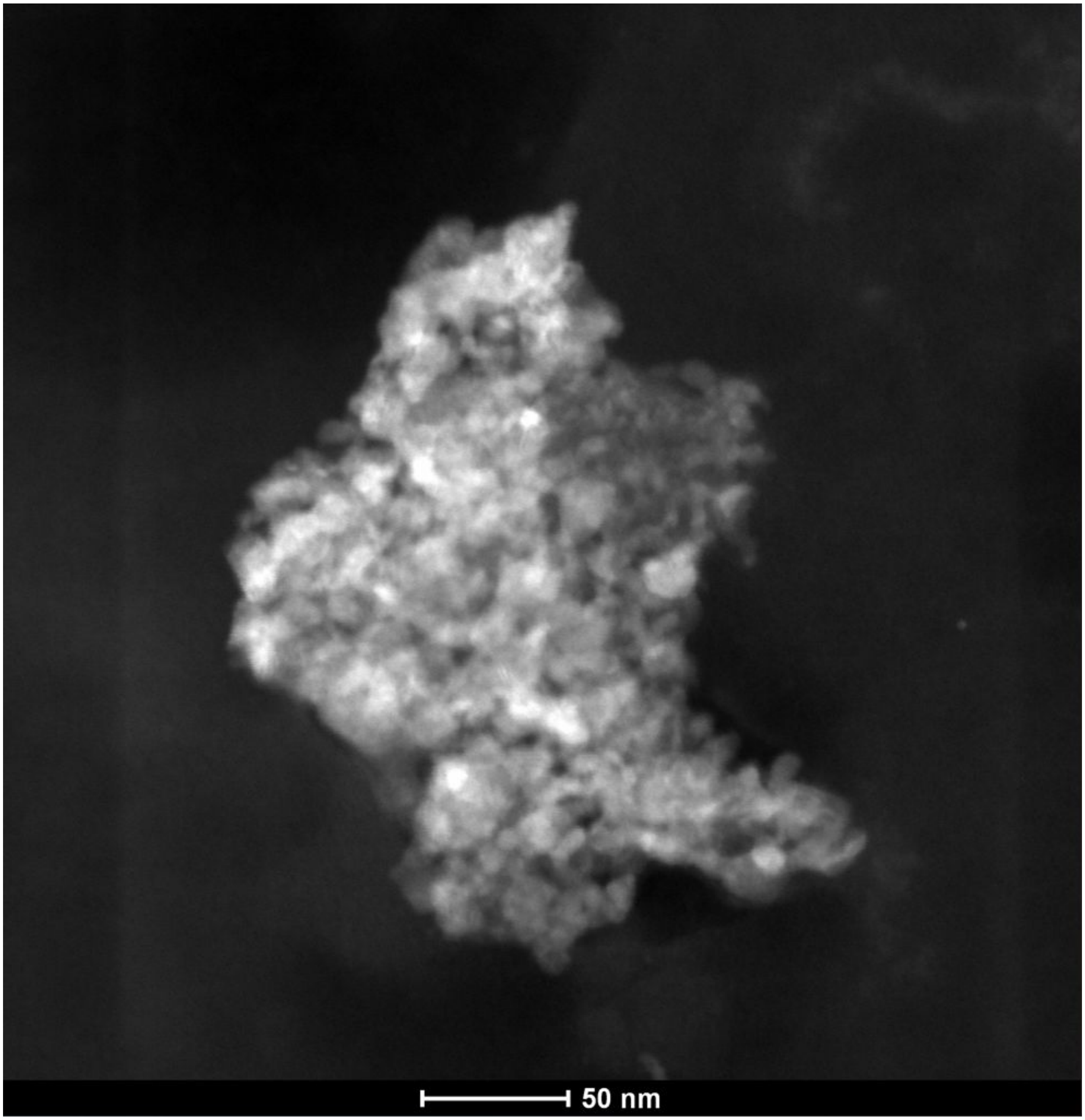

Figure S6. *Proposed first N-de-ethylation step of Rhodamine B under visible-light photocatalysis.* The scheme shows the conversion of Rhodamine B (m/z 326) to its de-ethylated derivative (m/z 282) through hydroxyl-radical attack on the diethylamino group. The lower mass spectrum (GC–MS, 40 min) confirms the presence of both ions at **m/z 326 and 282**, consistent with the partial loss of ethyl groups. Further details of the observed fragments are summarized in **Table S8** below.

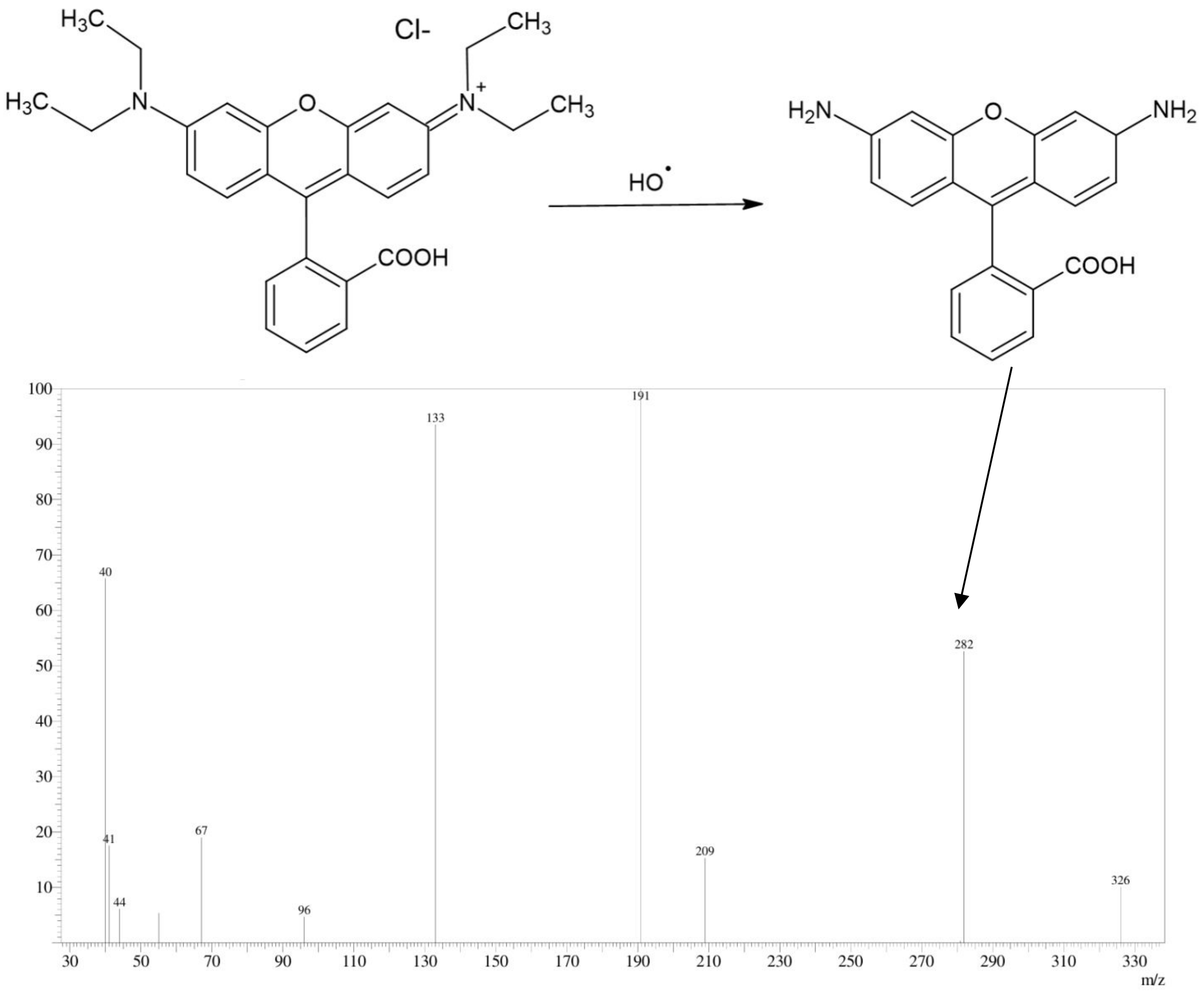

Table S2. GC–MS Evidence Supporting the N-de-ethylation Pathway of Rhodamine B

Sample / Time	Main peak RT (min)	Characteristic m/z (major ions)	Proposed assignment	Trend (40 → 300 min)	Interpretation / Relation to mechanism
GC01 (40 min)	≈ 30.82	191 (base); 133, 209, 282, 326	Rhodamine B with initial N-de-ethylation	— (start point)	Parent dye predominates; m/z 326 confirms RhB; co-appearance of 282 indicates onset of ethyl loss ($-C_2H_5$).

GC02 (300 min)	≤ 15	159, 173, 101, 85, 59, 43	Light fragments from xanthene core and diethylamino moiety	326 → disappears / low-m/z ions → increase	Advanced N-de-ethylation and chromophore cleavage; formation of low-mass aromatics and aliphatic fragments.
(Comparison)	—	Δ m/z ≈ 44 (326 → 282)	Loss of two ethyl groups (−2 × C_2H_5) ≈ N-di-de-ethyl RhB	Observed indirectly in mass pattern	Consistent with stepwise de-ethylation mechanism.

Note: m/z values obtained from GC–MS chromatograms of the 40 min (GC01) and 300 min (GC02) samples. Low-mass ions correspond to fragments formed after stepwise N-de-ethylation and partial chromophore cleavage.

Figure S7. Schematic representation of the photocatalytic reactor setup.

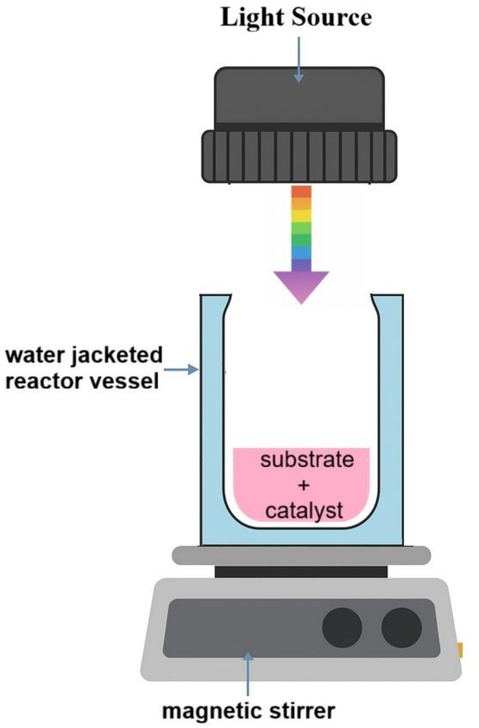

Figure S8. Rietveld refinement of the XRD pattern for the Fe_3O_4@TiO_2-CuO composite showing observed (obs), calculated (calc), background (bkg), and difference (diff) curves. Vertical markers indicate Bragg reflections for Fe_2O_3, Fe_3O_4, TiO_2-anatase, TiO_2-rutile and CuO.

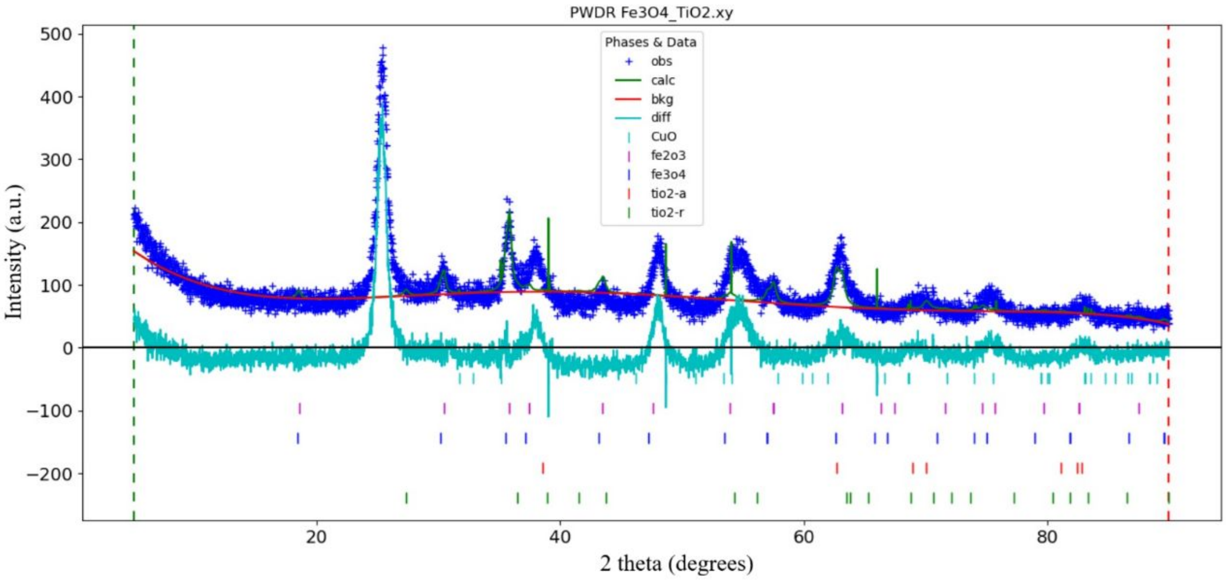

Figure S9. Room-temperature VSM curve of Fe_3O_4@TiO_2–CuO composite with an inset displaying the enlarged central region of the hysteresis loop.

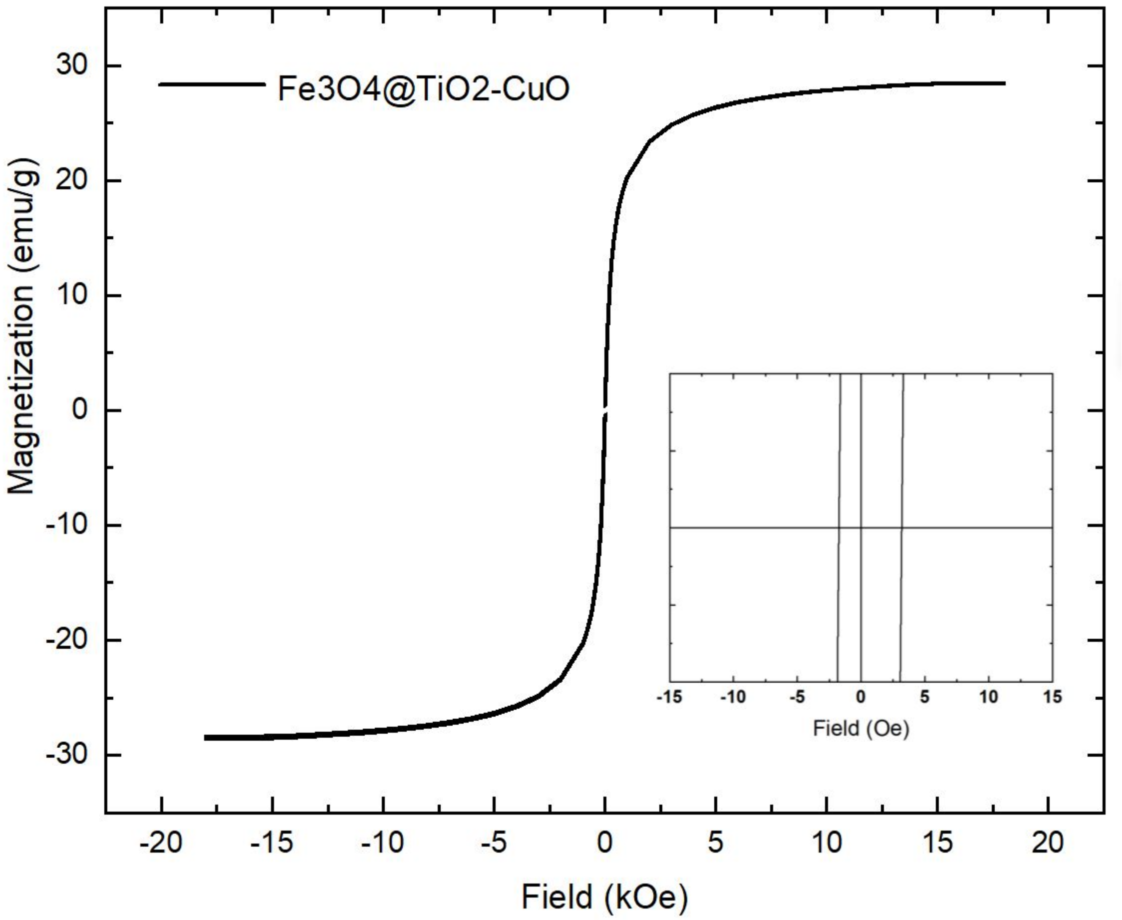

References

(1)Mohammadi-Aghdam, S.; Sarkhosh, B.; Tajoddin, N. N. Recyclable Fe3O4/SiO2/TiO2/Cu Nanocomposites: Synthesis, Characterization and Investigation of the Photocatalytic and Magnetic Property. *J. Mater. Sci.: Mater. Electron* **2017**, *28*, 9456–9463. https://doi.org/10.1007/s10854-017-6688-x.

(2) Predoana, L.; Petcu, G.; Preda, S.; Pandele-Cușu, J.; Petrescu, S. V.; Băran, A.; Apóstolo, N. G.; Costescu, R. M.; Surdu, V.-A.; Vasile, B. Ş. Copper-/Zinc-Doped TiO_2 Nanopowders Synthesized by Microwave-Assisted Sol–Gel Method. *Gels* **2023**, *9* (4), 267. DOI: 10.3390/gels9040267

(3) Rani, N.; Dehiya, B. S. Magnetically Recyclable Copper Doped Core-Shell Fe_3O_4@TiO_2@Cu Nanocomposites for Wastewater Remediation. *Environ. Technol.* **2021**, *43* (28), 4484-4492. DOI: 10.1080/09593330.2021.1954094

(4) Rafieezadeh, M.; Kianfar, A. H. Synthesis and Characterization of the Magnetic Submicrocube Fe_3O_4/TiO_2/CuO as a Reusable Photocatalyst for the Degradation of

Dyes under Sunlight Irradiation. *Environ. Technol. Innov.* **2021**, *23*, 101756. DOI: 10.1016/j.eti.2021.101756

(5) Vinosel, V. M.; Anand, S.; Janifer, M. A.; et al. Preparation and Performance of Fe_3O_4/TiO_2 Nanocomposite with Enhanced Photo-Fenton Activity for Photocatalysis by Facile Hydrothermal Method. *Appl. Phys. A* **2019**, *125* (5), 1-13. DOI: 10.1007/s00339-019-2622-9

(6) Dubey, M.; Kumar, R.; Srivastava, S. K.; Joshi, M. Visible Light Induced Photodegradation of Chlorinated Organic Pollutants Using Highly Efficient Magnetic Fe_3O_4/TiO_2 Nanocomposite. *Optik.* **2021**, *243*, 167309. DOI: 10.1016/j.ijleo.2021.167309

(7) Xu, Q.; Li, E.; Zhao, R.; et al. Preparation of Organic Porous Materials-TiO_2/Cu Composite with Excellent Photocatalytic Degradation Performances toward Degradation of Organic Pollutants in Wastewater. *J. Polym. Res.* **2020**, *27* (7), 186. DOI: 10.1007/s10965-020-02163-9

(8) Abbas, N.; Shao, G. N.; Imran, S. M.; et al. Inexpensive Synthesis of a High-Performance Fe_3O_4-SiO_2-TiO_2 Photocatalyst: Magnetic Recovery and Reuse. *Front. Chem. Sci. Eng.* **2016**, *10*, 405-416. DOI: 10.1007/s11705-016-1579-x

(9)Chishti, A. N.; Ma, Z.; Liu, Y.; Chen, M.; Gautam, J.; Guo, F.; Ni, L.; Diao, G. Synthesis of Highly Efficient and Magnetically Separable Fe_3O_4@C-TiO_2-Ag Catalyst for the Reduction of Organic Dyes and 4-Nitrophenol. *Colloids Surf. A.* **2021**, *631*, 127694. DOI: 10.1016/j.colsurfa.2021.127694

(10) Mortazavi-Derazkola, S.; Salavati-Niasari, M.; Amiri, O.; Abbasi, A. Fabrication and Characterization of Fe_3O_4@SiO_2@TiO_2@Ho Nanostructures as a Novel and Highly Efficient Photocatalyst for Degradation of Organic Pollution. *J. Energy Chem.* **2017**, *26* (1), 17-23. DOI: 10.1016/j.jechem.2016.10.015
